# Liraglutide mitigates dexamethasone-induced fatty acid synthase (FASN) and the cluster of differentiation36 (CD36) expression: a potential treatment for glucocorticoid-induced non-alcoholic fatty liver disease (NAFLD)

**DOI:** 10.1007/s00210-025-03789-6

**Published:** 2025-01-17

**Authors:** Dahshan Hassan Selim, Hamada Ahmed Mokhlis, Abdelrahman M. Elsayed, Abdel-Aziz S. Shatat, Salama Abdou Salama, Raed Shahat Ismail

**Affiliations:** 1 Department of Pharmacology and Toxicology, Faculty of Pharmacy (Boys), Al-Azhar University, Nasr City, 11651 Cairo Egypt; 2 Department of Pharmacy Practice, Faculty of Pharmacy, Sinai University-Kantara Branch, Ismailia City, 41636 Egypt; 3 Department of Ophthalmology and Visual Science, Havener Eye Institute, The Ohio State University Wexner Medical Center, Columbus, OH 43210 USA

**Keywords:** Non-alcoholic fatty liver disease, Dexamethasone, Fatty acid synthase, Cluster of differentiation 36, Liraglutide

## Abstract

The clinical use of dexamethasone (DXM) is associated with the development of non-alcoholic fatty liver disease (NAFLD). However, the mechanisms by which DXM-induced NAFLD is still incompletely known. Therefore, the current study aims to test the hypothesis that DXM-induced NAFLD is mediated by dysregulation of key genes involved in lipid metabolism and liraglutide (LG) can ameliorate these effects. The histopathological and biochemical analysis assessed the effects of DXM and/or LG in liver tissue. The computational analysis was performed to detect the glucocorticoid response elements (GRE) in the promotor regions of *FASN* and *CD36* genes. The effects of DXM and LG on the expression of *FASN* and *CD36* were determined by real-time quantitative reverse transcription PCR (RT-qPCR), western blot (WB) and immunohistochemical (IHC) analyses. The NAFLD induced by high-fat diet (HFD) and DXM was manifested by increased levels of liver enzymes, deterioration of histological architecture of the liver tissue and accumulation of fat droplets. Computational analysis revealed that the promotor regions of *FASN* and *CD36* harper several GRE. Most importantly, treatment with DXM decreased phosphorylated adenosine monophosphate-activated protein kinase (p-AMPK) levels, while LG upregulate it. In addition, treatment with DXM increased expression of *FASN* and *CD36*, whereas LG ameliorated these effects in a dose-dependent manner. DXM-induced NAFLD is mediated by upregulation of *FASN* and *CD36* expression which may be attributed to GRE. LG coupling with DXM mitigates this induction by downregulating *FASN* and *CD36* levels and therefore mitigates the development of NAFLD.

**Supplementary Information:**

The online version contains supplementary material available at 10.1007/s00210-025-03789-6.

## Introduction

Non-alcoholic fatty liver disease (NAFLD) in which the intrahepatic fat is more than 5% of the liver weight is characterized by triacylglycerol-rich macrovesicular and microvesicular lipid droplets within the hepatocytes. Generally, normal aggregation of triglycerides (TG) within the liver tissue is considered hepatoprotective; however, over-stockpiling of TG may drive metabolic dysfunction, inflammation, nonalcoholic steatohepatitis (NASH), fibrosis, cirrhosis, or even progress to hepatic carcinoma. Normally, the liver fatty acids (FAs) stored are the result of equilibrium between FAs inflow through food intake and adipose tissue lipolysis, de novo lipogenesis (DNL), and elimination of FAs via β-oxidation or VLDL-C assembly and excretion (Vancells Lujan et al. 2021).

Dexamethasone (DXM) is one of the most popular synthetic anti-inflammatory glucocorticoids. Pharmacologically, DXM is 25 times more potent than cortisol in its glucocorticoid effect. It is used extensively for patients with allergies, asthma, autoimmune diseases, sepsis, cancers, and metabolic disorders. Unfortunately, nearly 90% of patients experience a range of side effects including hyperglycemia due to increased insulin resistance and impaired glucose tolerance that may eventually lead to the development of NAFLD (Fong and Cheung 2013). Aside from its hyperglycemic effect, DXM activates hepatic lipogeneses by increasing the expression of FASN and CD36, two fundamental genes responsible for fatty acids synthesis and uptake, respectively. Therefore, DXM could be used to establish models of NAFLD in rats (Malkawi et al. 2018; Rahimi et al. 2020). Subsequently, there is a pressing need to develop therapeutic measures to truncate DXM-induced fatty liver.

FASN is an enzyme that catalyzes the last step in FAs biosynthesis; thus, it is believed to be a significant determinant of the maximal hepatic capacity to generate FAs by DNL. Patients with NASH express higher levels of FASN and sterol regulatory element-binding proteins (SREBPs), the major transcriptional factors in lipogenic gene expression including FASN. Besides, hepatic FASN level was towered in vivo in a murine model of NASH. Therefore, the FASN level is considered a diagnostic score and therapeutic target for improving NAFLD (Hu et al. 2021).

The multi-ligand receptor CD36 is a membrane glycoprotein found in hepatocytes with a high affinity for FAs to orchestrate FAs cellular uptake. Besides, CD36 is a widespread transcription aim for multiple ligand-sensing and lipogenic transcription factors. Even though CD36 expression level is not high in liver cells under physiological conditions, it is highly induced when lipid overload or nuclear cell activation occurs (Glatz and Luiken 2017). It has been shown that levels of CD36 are higher in patients with NAFLD than in normal subjects, pointing to a potential role of CD36 in lipid metabolism; consequently, its transcriptional regulators pathways are considered therapeutic targets for treating NAFLD (Mundi et al. 2020).

Liraglutide (LG) is an acylated synthetic long-acting agonist of the glucagon-like peptide-1 (GLP-1) receptor bound to the membrane-bound enzyme adenylate cyclase (AC) catalyzing the conversion of cyclic adenosine triphosphate (cATP) to cyclic adenosine monophosphate (cAMP). The increase in the cAMP stimulates the glucose-dependent release of insulin, inhibits the glucose-dependent release of glucagon, and slows gastric emptying, and suppresses energy intake (Knudsen and Lau 2019). Liraglutide increases genes and proteins responsible for peroxisomal fatty acid β-oxidation, reverses diet-induced metabolic dysfunction, restores insulin sensitivity, improves hepatic lipid handling, and recovers liver function (Mells et al. 2012). Besides, LG has shown a significant impact on body weight and clinical, biochemical, and histological markers of fatty liver and fibrosis in patients with NAFLD. Therefore, LG could be a weapon for the treatment of both diabetes mellitus and NAFLD (Nevola et al. 2023).

The previously mentioned findings provided us with a rationale to hypothesize that LG could attenuate DXM-induced hepatic steatosis via downregulating CD36 and FASN signaling pathways. Subsequently, this study aims to estimate the alleviating effect of LG on DXM-induced NASH and investigate the role of CD36 and FASN in mediating this action.

## Materials and methods

### Ethical approval and animal care

Post-approval of the Institutional Animal Ethics Committee of the Faculty of Pharmacy, Al-Azhar University which follows the Guide for the Care and Use of Laboratory Animals published by the US National Institutes of Health (NIH publication number 85-23, revised 1996). The ethical approval number is Azhar-Pharmacy-2023-11-001. The experimental animal facility of the Nile for Pharmaceuticals & Chemical Industries (Cairo, Egypt) was the source for purchasing the 2-month-old and approximately 180–200 g weight adult Wistar albino male rats used in the study. Two rats in each cage were kept for acclimation in controlled housing conditions for 2 weeks (room temperature 25± 2°C, humidity (50–70%) and 12/12 h dark-light cycles) and kept free on a standard rodent diet (kcal%:10% fat, 20% protein, and 70% carbohydrate; 3.85 kcal/g). Rats were given ad libitum access to food and water.

### Experimental design and treatment protocol


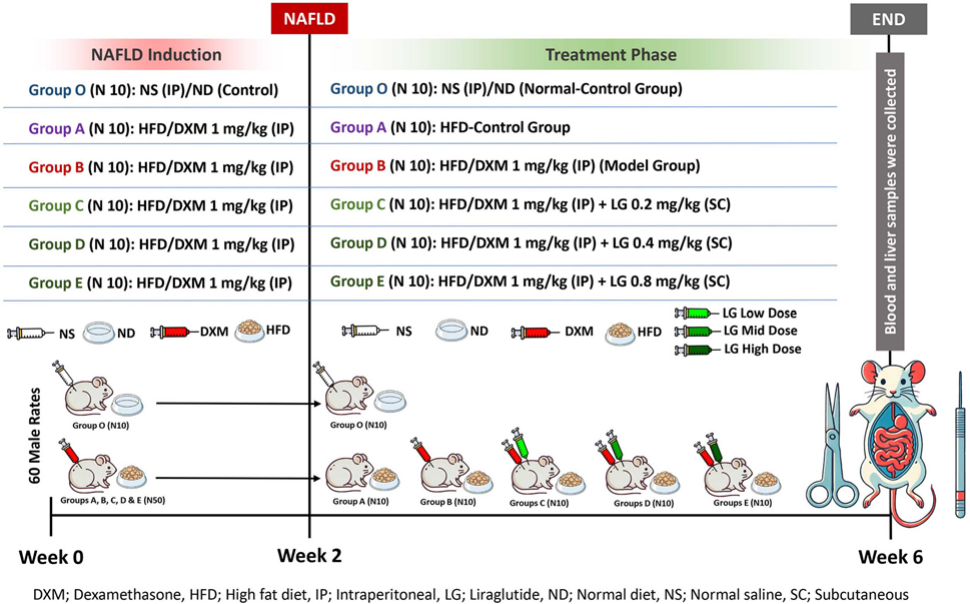


#### NAFLD induction

Sixty acclimatized male rats comparable in age and weight were randomly divided into six groups (*n*=10): O, A, B, C, D, and E. The animals were labelled and weighed. The DXM treatment regimen was initially chosen based on a previous study (Feng et al. 2020) and modified according to our preliminary pilot trials feedback. The high-fat diet (HFD) with a composition of 45% fat, 37% carbohydrate, and 18% protein was purchased from Uccma for Animal Feed (El Khanka, Abu Zaabal, Egypt) and matched the formulation described by (Cheng et al. 2018). It was used as the standard food throughout the experiment duration in all groups except the control group (O). The entire study treatment period lasted for 6 weeks; the control group (O) received no treatment over the 6 weeks and was kept on the normal standard diet. During the NAFLD induction phase (first 2 weeks) rats in groups A, B, C, D, and E received HFD and were intraperitoneally (IP) injected with DXM 1 mg/kg once daily to induce NAFLD (dexamethasone 8mg/2 ml Amp, Amriya for Pharmaceutical Industries Alexandria, Egypt), which was diluted with water for injection (El-Nasr Pharmaceutical Chemicals Company, Abou-Zaabal, Egypt). At the end of the first 2 weeks, animals were weighed. The animals were fasted for 12 h overnight, and the blood samples from each rat (2 ml) were withdrawn via the tail vein blood sample collection technique in which the animal was made comfortable in a continuously alcohol-sterilized restrainer while maintaining the temperature around 24 to 27 °C. Local anesthetic cream was applied on the surface of the tail 30 min before the experiment. A 23G needle was inserted into the blood vessel and blood was collected using a capillary tube. After completing blood collection, pressure/silver nitrate ointment/solution was applied to stop the bleeding (Parasuraman et al. 2010).

#### The treatment regimen for rats

During the next 4 weeks, all groups, except the control group (O), were maintained on HFD: Group A, referred to as the HFD-control (or DXM-withdrawal), discontinued DXM treatment and did not receive any treatment; group B, referred to HFD/DXM-treated group (or model group), continued to receive DXM 1 mg/kg IP daily, group C continued to receive DXM 1 mg/kg IP plus LG 0.2 mg/kg (low-dose = LD) (Novo Nordisk, Denmark) subcutaneous (SC) once daily, group D continued to receive HFD/DXM 1 mg/kg IP alongside with LG 0.4 mg/kg (mid-dose = MD) SC once daily, and group E received HFD/DXM 1 mg/kg IP alongside with LG 0.8 mg/kg (high-dose = HD) SC once daily. LG treatment regimens were convergently chosen according to the previous study (Zhang et al. 2020) and modulated according to our pilot experiments observations. At the end of the treatment period, just before the euthanizing of rats, animals were reweighed and fasted 12-h overnight, and the blood samples from each rat (2 ml) were withdrawn via tail vein technique. The collected blood samples were processed for the previously mentioned biochemical markers analysis and the per cent change of the animal’s body weight was calculated as follows:

$$Percent\;change\;of\;the\;body\;weight\;=\;\frac{body\;weight\;at\;the\;end- initial\;body\;weight}{initial\;body\;weight}\;\times\;100$$

### Biochemical analysis

Blood samples were allowed to clot at room temperature for 15–30 min. The sera were separated by centrifugation at 1500 x g for 10 min in a refrigerated centrifuge. The separated sera were stored at – 80 °C until used for biochemical analysis. The clinical chemistry analyzer Spinlab Photometer was used after standardization for the quantitative colorimetric assay for alanine transaminase (ALT), aspartate aminotransferase (AST), fasting blood sugar (FBS), total cholesterol (TC), low-density lipoprotein cholesterol (LDL-C), very low-density lipoprotein cholesterol (VLDL-C), and triglycerides (TG), and high-density lipoprotein cholesterol (HDL-C) according to the standard manufacturer’s instructions inserted in their kits (Bio-Med Company, Cairo, Egypt).

### Liver sample preparation for assay

Rats were euthanized by cervical dislocation technique according to standard animal euthanasia method guidelines developed in 2010 by the Canadian Council on Animal Care (CCAC). The livers from the different treatment groups were removed and examined macroscopically rapidly and then divided into two parts; the first portion was immediately preserved in liquid nitrogen for subsequent analysis of FASN and CD36 expression by Western blotting (WB) and real-time quantitative reverse transcription PCR (RT-PCR), while the second portion was fixed in a 10% buffered formalin solution for 48 h to enable immunohistochemical (IHC) and histopathological examinations. The tissues were then dehydrated in ascending grades of ethanol, cleared in xylene, embedded in paraffin, and sectioned at a thickness of 4–5μm. The same dehydrated sectioned slides were processed for IHC for detecting FASN and CD36 expression, histopathologically for detecting liver tissue deterioration markers and to estimate lipids in the tissues of the liver.

### Examination of liver tissue deterioration

The liver tissue was prepared for histopathological assessment where the previously formalin-fixed slides were dehydrated and stained by a combination of hematoxylin and eosin (H&E) according to the previously described method. Briefly, after tissue collection and fixing, they were typically dehydrated and embedded in melted paraffin wax; the resulting block is mounted on a microtome and cut into thin slices. The slices were affixed to microscope slides at which point the wax is removed with a solvent and the tissue slices attached to the slides are rehydrated and are ready for staining. Alternatively, H&E stains were typically frozen, cut on a cryostat (a microtome that cuts frozen tissue), fixed in alcohol, and then stained. The hematoxylin-stained cell nuclei a purplish blue, and eosin stained the extracellular matrix and cytoplasm pink (Al-Dulaimi et al. 2018; Dapson and Horobin 2009). The qualitative lesion score of the liver tissues in different experimental groups was tabled and scored according to the American Association for the Study of Liver Diseases (AASLD) Clinical Single Topic Conference on NASH held in 2002 (Takahashi and Fukusato 2014).

### Estimation of oil droplets in liver tissue

The Oil Red O (ORO) staining method for the quantitative lipid droplet detection in the liver tissues was processed on the formalin-fixed slides as previously prescribed by Stotz et al. After floating in the water and attaching the slides to albumenized slides, they were allowed to dry thoroughly (one to 2 h). The slides are then dipped with a 60% isopropyl alcohol and oil red O working solution for 20 min. A 60% isopropyl alcohol thereafter was added to the slides. Then, the slides were rinsed with four changes of distilled water and counterstained with acidified Lillie-Mayer hematoxylin for 1 min followed by rinsing in three changes of distilled water. Blue hematoxylin in 0.3% sodium borate is then added for 15 s, followed by rinsing with four changes of distilled water and mounting with glycerin jelly. The stained liver section slides were placed on the microscope stage, and the 20X objective lens was used to visualize the stained lipid droplets in the liver tissue (Stotz et al. 1986). Then, the high-quality digital images of representative fields of view were captured by the LABOMED Trinocular inverted phase contrast microscope model TCM400 microscope, and the Atlas 16MP Cmos USB Camera software (LABOMED, USA). The magnification power was 20x, and the scale bar was 50 µm.

The captured images were transferred to the computer software (LABOMED camera software, USA), and the images were opened by the image analysis software (Atlas 16MP Cmos USB Camera software). Using a scale of 50µm, the software was calibrated to ensure accurate measurements. The software’s tools were used to outline and measure the stained lipid droplets in the liver tissue. The color threshold was adjusted to isolate the stained areas accurately. Finally, the data were recorded, including the examined area, standard deviation, and percentage of the area of stained lipid droplets, their number, and the minimum and maximum threshold. The results are summarized in tabular format, including average measurements from multiple fields of view.

### Measurement of inflammatory markers in liver tissue

Tumor necrosis factor-α (TNF-α), a key pro-inflammatory cytokine involved in liver inflammation induced by lipid accumulation in hepatic tissue, was quantitatively measured using the corresponding enzyme-linked immunosorbent assay (ELISA) kits (MyBioSource, San Diego, California, USA). Sample preparation and assay procedures were performed strictly according to the manufacturer’s instructions (Catalog number: MBS175904). Briefly, liver samples were homogenized and centrifuged, and the resulting supernatant was collected for analysis. The assay involved adding specific capture and detection antibodies to the wells, followed by incubation to ensure binding specificity to TNF-α. Optical density (OD) readings were measured at 450 nm using a microplate reader, and the concentration of TNF-α was calculated based on a standard curve generated from known concentrations (Ceyhan et al. 2018). The concentration of TNF-α was originally measured in pg/mL and subsequently expressed as pg/mg liver, assuming an initial liver weight of 1 mg/mL in homogenate preparation.

### Exploring glucocorticoid receptor binding sites in FASN and CD36 via in silico analysis

In-silico analysis was performed to investigate the potential transcriptional regulation of FASN and CD36 genes by glucocorticoid receptors. Promoter sequences for both genes were retrieved from the Eukaryotic Promoter Database (EPD) (https://epd.epfl.ch) using the transcription start site (TSS) as a reference point. The promoter regions analyzed spanned −2000 to +100 nucleotides relative to the TSS.

The AliBaba2.1 software (http://gene-regulation.com/pub/programs/alibaba2/) was utilized to predict transcription factor binding sites, specifically focusing on glucocorticoid response elements (GREs). This software identifies binding sites by comparing the promoter sequences with known consensus sequences for transcription factors. Predicted GRE half-sites were highlighted and mapped to the promoter regions of both FASN and CD36.

### Detecting FASN and CD36 expression in liver tissue

Real-time quantitative reverse transcription PCR (RT-qPCR) was performed to evaluate the expression levels of FASN and CD36 mRNA. Total RNA was extracted from tissue lysates using the Direct-zol™ RNA MiniPrep Plus Kit (Zymo Research Corp., USA). The RNA quantity and quality were assessed using a Beckman dual spectrophotometer. For extraction, TRI Reagent^®^ was added to the lysate, followed by ethanol to facilitate binding to the Zymo-Spin™ IIICG Column. The mixture was processed through the column, washed, and eluted to yield high-quality RNA suitable for downstream applications. The SuperScript™ IV One-Step RT-PCR Kit was employed to synthesize cDNA and amplify it in a single reaction using real-time PCR (Applied Biosystems). The reaction mixture included SuperScript™ IV RT Mix, 2X Platinum™ SuperFi™ RT-PCR Master Mix, and gene-specific primers for FASN and CD36. The primers were predesigned and ordered from Sigma-Aldrich™ (USA) using RefSeq IDs NM_017332 and NM_031561 and the primer sequences listed in Table 1. The one-step protocol ensured efficient reverse transcription and PCR amplification within the same tube, thus enhancing sensitivity and reducing handling. For relative quantification of gene expression, the Cycle threshold (Ct) values of target genes, FASN and CD36, were normalized against the housekeeping gene GAPDH. The relative expression levels were determined using the delta-delta Ct (ΔΔCt) method and presented as 2^(-ΔΔCt), allowing comparison across experimental conditions (Huang et al. 2020).

**Table 1:** The sequences of primers used for real-time quantitative PCR

	Sense primer (5′−3′)	Anti-sense primer (5′−3′)
*FASN*	AAAAGGAAAGTAGAGTGTGC	GACACATTCTGTTCACTACAG
*CD36*	AAGGAATTTGTCCTATTGGG	GAGACTTCTCAACAAAAGGTG

Western blotting technique estimated the liver tissue expression of FASN and CD36 enzyme as previously described (Eisa et al. 2023; Shatat et al. 2024). The Bio-Rad DC Protein Assay (Bio-Rad Laboratories, USA) measured protein in the liver tissue homogenates. Proteins in each sample were denatured at 95 °C for 5 min in 5× Laemmli buffer containing 100 Mm Dithiothreitol (DTT). By loading equal amounts of protein (50–100 µg per lane) and separated by sodium dodecyl sulfate-polyacrylamide gel electrophoresis at 120 V through 6% stacking gel and 8% or 10% resolving gel using Mini PROTEAN Tetra system unit (BioRad, USA, serial no. 552BR) for approximately 2 h and then transferred to polyvinylidene fluoride (PVDF) membranes using mini-gel wet-transfer XCell II™ Blot Module (Thermo-fisher scientific, Invitrogen, USA, Cat. No. EI9051) at 25 V for 90 min. Then, we blocked transferred PVDF membranes by incubation in TBS buffer containing 0.1% Tween and 5% BSA (TBST) for 1 h at 4 °C. Thereafter, membranes were incubated with either Anti-CD36 Primary Rabbit monoclonal antibody (1:1000) or Anti-FASN Primary Rabbit monoclonal Antibody (1:1000) overnight at 4° C. For detection of AMPK activation, membranes were further incubated with Anti-AMPKα Primary Rabbit monoclonal antibody (1:1000) and Anti-phospho-AMPKα (Thr172) Primary Rabbit monoclonal antibody (1:1000) overnight at 4 °C. Then, they were washed with TBST buffer and incubated for 1 h at RT with Secondary Antibody Solution Alkaline Phosphatase-Conjugated (Anti-Rabbit) (Thermo-fisher Scientific, Invitrogen -USA). Following four washes with TBST, the membrane-bound antibody-specific bands were visualized by using Novex™ AP Chromogenic Substrate (BCIP⁄ NBT) (Thermo-fisher Scientific, Invitrogen, USA), and the intensity of the bands was quantified by ImageJ/NIH software. Equivalent protein loading for each lane was confirmed by stripping and reblotting membranes at 4 °C against rabbit monoclonal anti-ß-actin antibody (1:1000).

In addition, immunohistochemical analysis was performed for FASN and CD36 immunoreactivity assessment as described by Magaki et al. The formalin-fixed, paraffin-embedded tissue sections from livers were treated by heat-induced epitope retrieval technique (temperature 50 °C for up to 60 min). Concentrated primary antibodies FASN (Recombinant Fatty Acid Synthase (C20G5) Rabbit mAb #3180, Cell Signaling Technology^®^, USA) and CD36 (Recombinant CD36 (E8B7S) Rabbit mAb #28109, Signaling Technology^®^, USA). Subsequently, the concentrated primary antibodies were diluted, incubated and auto-stained with a high-sensitivity visualization system (Envision™ FLEX, High pH, and Link system) in the autostainer Link 48 in which the software had been pre-programmed according to the manufacturer’s recommendations provided in the package insert of the concentrated primary antibodies. The sectioned slides were examined using a light microscope to assess the expression of both FASN and CD36 in all groups. Six images for each slide were obtained (x400). Immunoreactivity was evaluated by estimating the area percentage of the positive brown immunostained cells, using computed image analysis by Leica Qwin software 500, Germany (Magaki et al. 2019).

### Statistical analysis

Graph Pad Prism software (version 9.0.2, San Diego, CA, USA) was used for statistical analyses. All values are shown as the mean ± S.D. The acquired groups’ data were analyzed using one-way ANOVA followed by the Tukey-Kramer test. *P<*0.05 was considered to be statistically significant. Correlation statistical analysis was performed to investigate the correlation between lipid expressions obtained from the oil red O method and both FASN and CD36 expression gained from immunohistochemical and western blot techniques.

## Results

### Liraglutide reverses DXM-induced weight gain and changes in liver architecture

**Fig. 1:**
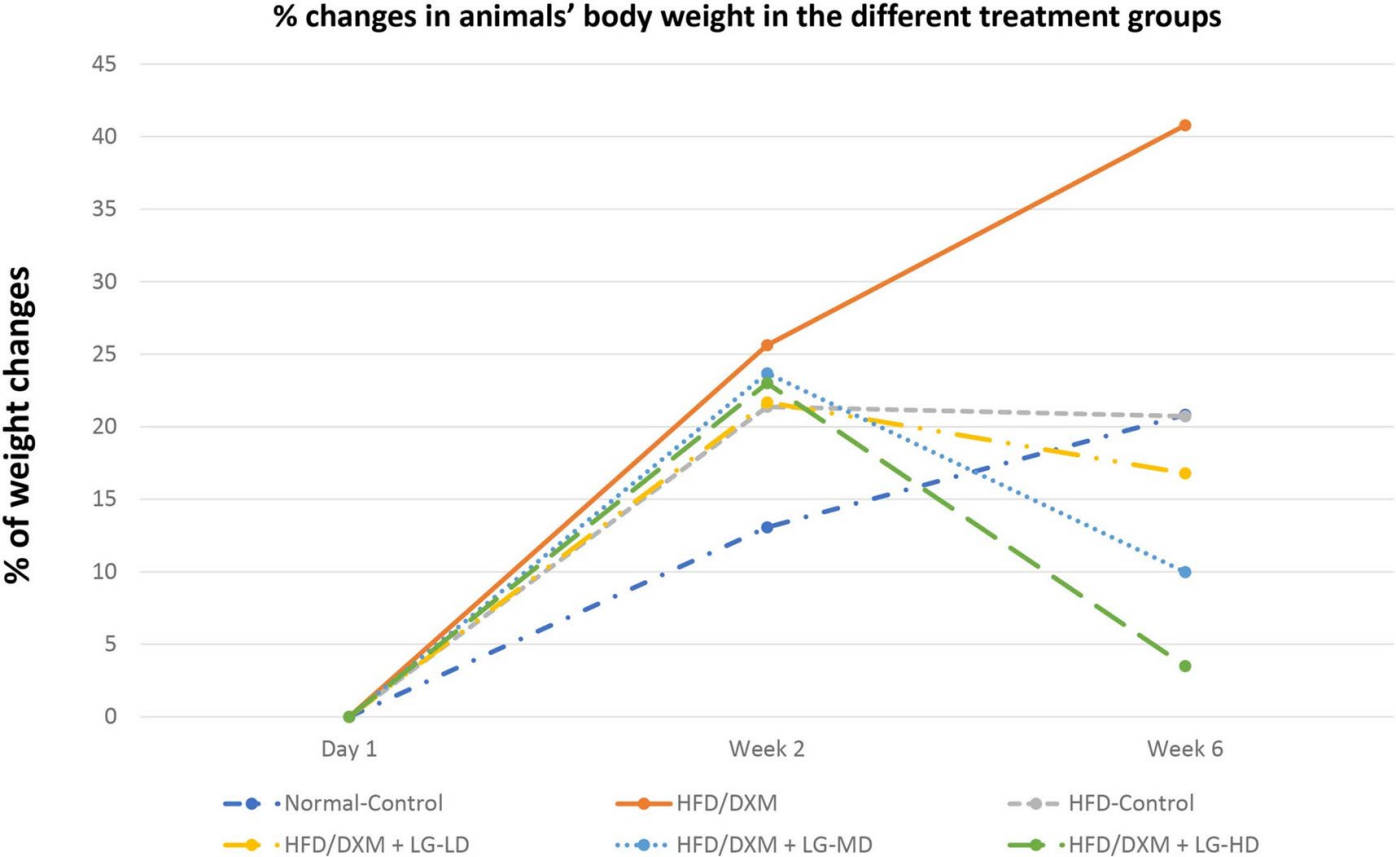
Percent changes in animals’ body weight. A Significant weight reduction in groups treated with HFD/DXM plus LG at different doses compared to the HFD/DXM-treated group. DXM, dexamethasone; HFD, high-fat diet; LG, liraglutide; LD, low-dose 0.2 mg/kg; MD, moderate-dose 0.4 mg/kg; HD, high-dose 0.8 mg/kg

Given the fact that glucocorticoids treatment is associated with an increase in body weight, we set out to establish dexamethasone-induced fatty liver model in male rats. Our results revealed that administration of DXM (1 mg/kg/day, IP) daily for 2 weeks led to a remarkable increase in the % of total body weight compared with the control group (25.62±4.73 *vs* 13.08±3.13, *P* < 0.05). Nonetheless, withdrawal of DXM treatment for 4 weeks resulted in returning of body weight to approximately normal levels (20.73±5.28 *vs* 20.82±4.32, *P* > 0.05). Liraglutide (LG), a glucagon-like peptide 1 analog, has been shown to reduce body weight and ameliorate liver metabolic dysfunction in multiple experimental and clinical studies (Nevola et al. 2023). To determine whether LG affects weight gain induced by DXM, we tested three different doses of LG and observed that coadministration DXM (1 mg/kg/day, IP) with LG (SC 0.2, 0.4, 0.8 mg/kg/day) is associated with dose-dependent amelioration of DXM-induced weight gain compared with DXM-treated group (16.78±2.46, 9.99±2.98 and 3.46±1.45, *respectively vs* 40.78±6.58, *P* < 0.05) (Fig. 1).

**Fig. 2:**
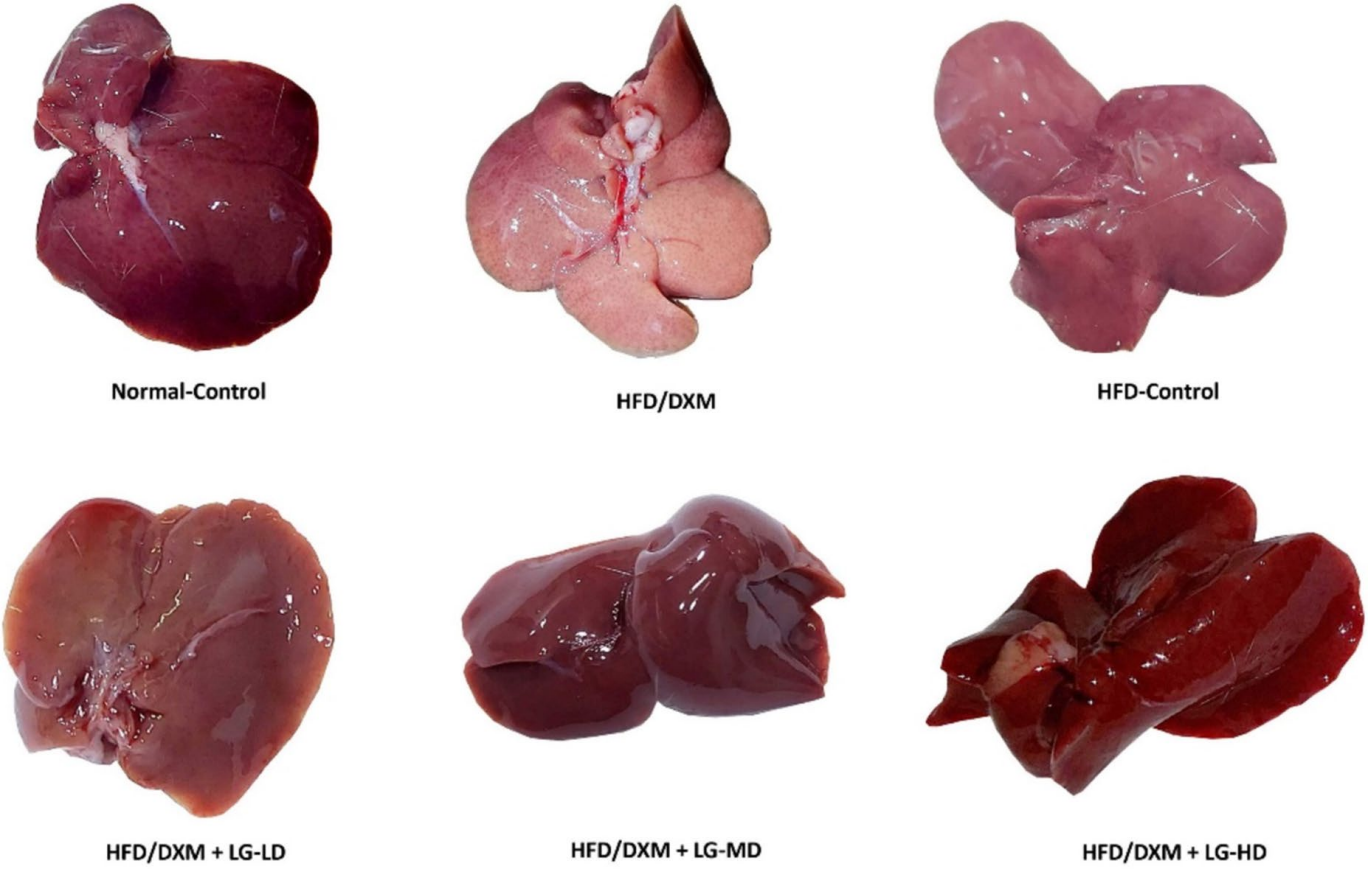
A gross liver examination of the different experimental groups at the end of the DXM and LG treatment period. The liver appearance in the HFD/DXM-treated group shows typical features of the fatty liver (yellow color, blunt with a crispy edge, and abundant fat droplets) compared with the liver in the HFD-control group and groups treated with HFD/DXM coupled with different doses of LG. DXM, dexamethasone; HFD, high-fat diet; LG, liraglutide; LD, low-dose 0.2 mg/kg; MD, moderate-dose 0.4 mg/kg; HD, high-dose 0.8 mg/kg

The macroscopic appearance is one of the most distinguishing characteristics of fatty liver. Therefore, we examined the gross features of livers following administration of DXM and/or LG. Our results demonstrated that rats treated with HFD/DXM exhibited the typical features of fatty liver in terms of the yellow color, blunt with a crispy edge, and abundant fat droplets. Meanwhile, the macroscopic examination of livers in the control group, HFD-control group, and HFD/DXM plus LG groups showed few or absence of fat droplets, dark red with sharp livers, and flexible texture edges (Fig. 2). Collectively, these data suggest that DXM induces fatty liver and LG treatment remarkably mitigates the development of fatty liver in a dose-dependent manner.

### Liraglutide alleviates DXM effects on liver function, lipid profile, and blood glucose level

To investigate whether the effect of DXM and/or LG on the macroscopical appearance of the liver is associated with alteration in liver function, lipid profile, and blood glucose level, we measured ALT, AST, FBS, TC, LDL-C, VLDL-C, TG, and HDL-C in different treatment groups. Our data demonstrated that treatment with DXM for 2 weeks resulted in a significant increase in ALT, AST, FBS, TC, LDL-C, VLDL-C, and TG and decrease in HDL compared with the control group. Discontinuation of DXM treatment restored the serum levels of the investigated parameters to nearly normal levels. Likewise, LG treatment combined with DXM resulted in a significant decrease in ALT, AST, FBS, TC, LDL-C, VLDL-C, and TG serum levels compared with DXM monotherapy (*P* < 0.05). In contrast, the levels of HDL-C in rats received DXM/LG dual therapy were significantly higher than those received DXM alone (*P* < 0.05, Table 2). Interestingly, the effect of LG treatment on liver function parameters, lipid profile, and serum glucose level seems to be dose dependent; high dose of LG resulted in pronounced response than moderate and low doses.

**Table 2:** The effect of DXM and/or LG on liver function parameters, lipid prolife, and serum glucose level

Biochemical markers in different experimental groups after the end of the experimental study (mean ± SD)								
Groups	ALT	AST	FBS	TC	LDL	VLDL-C	TG	HDL-C
Normal-control	27.9±5.78	72.22±15.49	76.3±5.85	107.8±6.7	46.78±3.11	13.69±1.65	72.44±3.05	48.5±6.31
HFD/DXM	82.8±3.61^a^	201.7±11.19^a^	181.2±8.11^a^	179.2±11.6^a^	88.78±6.38^a^	20.53±1.36^a^	198.6±7.89^a^	17.7±2.67^a^
HFD-Control	42.4±4.03	94.81±9.23	122.1±3.54	119.9±7.88	50.56±6.88	14.01±2.11	125.4±3.13	42.1±4.63
HFD/DXM/LG-LD	46.6±1.51^a,b^	102.6±7.64^a,b^	106.7±6.40^a,b^	94.22±8.35^a,b^	41.22±4.29^a,b^	12.31±1.04^a,b^	116.3±3.61^a,b^	54±2.21^a,b^
HFD/DXM/LG-MD	35.4±2.32^a,b,c^	75.51±8.58^a,b,c^	91.3±4.64^a,b,c^	79.89±8.42^a,b,c^	32.89±3.66^a,b,c^	9.18±0.74^a,b,c^	100.1±1.27^a,b,c^	61.9±4.7^a,b,c^
HFD/DXM/LG-HD	27±1.49^a,b,c,d^	63.96±5.57^a,b,c^	81.3±3.86^a,b,c,d^	65±4.66^a,b,c,d^	24.33±3.50^a,b,c,d^	7.65±0.72^a,b,c^	85.89±2.62^a,b,c,d^	77.2±4.52^a,b,c,d^

^“a,b,c,d”^ represents a significant difference (*P*< 0.05) from control, DXM, DXM + LG-LD, and DXM + LG-MD, respectively. *DXM* dexamethasone, *LG* liraglutide, *LD* low dose (0.2 mg/kg), *MD* moderate dose (0.4 mg/kg), *HD* high dose (0.8 mg/kg)

### Liraglutide improves DXM-induced histopathological changes in the liver

**Fig. 3:**
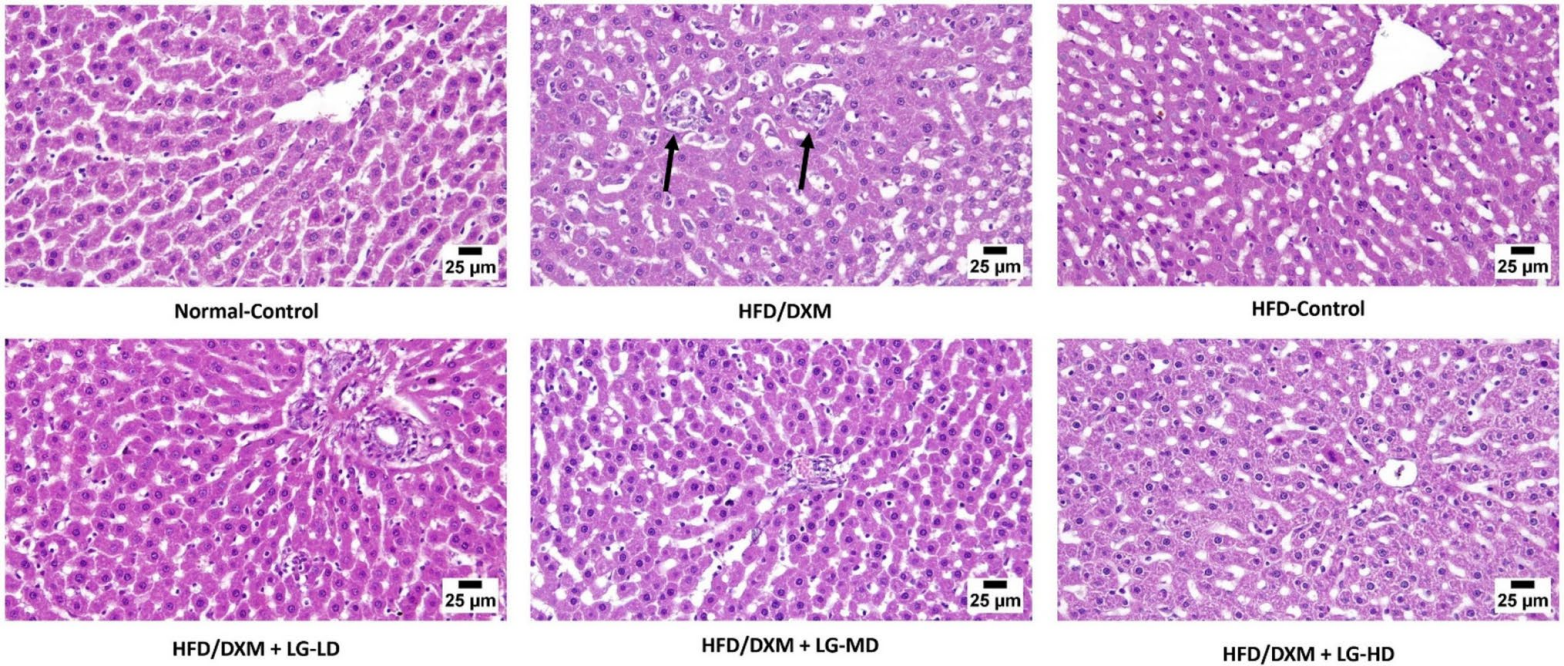
Photomicrograph of liver tissue section stained with hematoxylin and eosin (H&E) in the different experimental groups at the end of the experimental study. Images are captured at higher magnification, and the scale bar is 25µm. The normal structure of hepatic parenchyma without any detectable alterations in the control group. Withdrawal of DXM treatment after induction of fatty liver in HFD-control group exhibited the normal structure of liver tissue as well. Diffuse hepatocellular vacuolation, marked mononuclear inflammatory cells infiltrating the hepatic parenchyma with hepatocellular necrosis, and Kupffer cells hyperplasia (black arrows) were noticed in the examined sections from the HFD/DXM-treated group. Liver sections from HFD/DXM-treated rats coadministered with LG at different doses were normal except for a sporadic case that showed a focal area of hepatocellular necrosis with mild inflammatory cell infiltration. DXM, dexamethasone; HFD, high-fat diet; LG, liraglutide; LD, low-dose 0.2 mg/kg; MD, moderate-dose 0.4 mg/kg; HD, high-dose 0.8 mg/kg

Histopathological changes are reliable markers for fatty liver. Thus, we investigated the effect of DXM and/or LG on histopathological features and tissue architecture. At the end of the experimental study, histopathological examination of the liver tissue revealed a normal structure of hepatic parenchyma without any detectable alterations in the normal-control group and DXM-withdrawal group. Nevertheless, liver tissues from DXM-treated group exhibited diffuse hepatocellular vacuolation, remarkable infiltration of mononuclear inflammatory cells along with hepatocellular necrosis and an increase in Kupffer cells. On the other hand, liver sections retrieved from DXM + LG treated group showed normal histological feature except for a sporadic case with mild inflammatory cell infiltration (Fig. 3). The qualitative lesion score in various experimental groups showed that LG combined with HFD/DXM-treated rats had minimal inflammatory and deterioration markers. Despite the absence of a statistically significant difference in the liver deterioration markers among the groups treated with DXM + LG, our results demonstrated a significant diminution of these markers in DXM + LG groups compared to DXM-treated group (Table 3).

**Table 3:** Qualitative lesion score of liver tissues in different experimental groups

Markers	Normal-Control	HFD-Control	HFD/DXM	HFD/DXM + LG-LD	HFD/DXM + LG-MD	HFD/DXM + LG-HD
Groups						
A) Hepatocellular vacuolation	-	-	+++	-	-	-
B) Portal inflammation	-	-	+++	+	+	+
C) Hepatocellular necrosis	-	-	+++	-	-	-
D) Kupffer cell activation and hyperplasia	-	+	+++	+	-	-

(-) absent, (+) mild, (++) moderate, (+++) severe

The control group received no treatment over the six weeks, other groups were treated with DXM 1mg/kg for the first two weeks, after that, during the next four weeks: HFD-control group kept on HFD without treatment, other four groups kept on HFD with treatments: DXM-treated group continue to receive DXM 1 mg/kg, other groups continue to receive DXM 1 mg/kg plus LG at different three doses: LD (low-dose, 0.2 mg/kg), MD (moderate-dose, 0.4 mg/kg), and HD (high-dose 0.8 mg/kg); DXM, dexamethasone; LG, liraglutide

### Liraglutide attenuates the effect of DXM on lipid accumulation in liver tissues

**Fig. 4:**
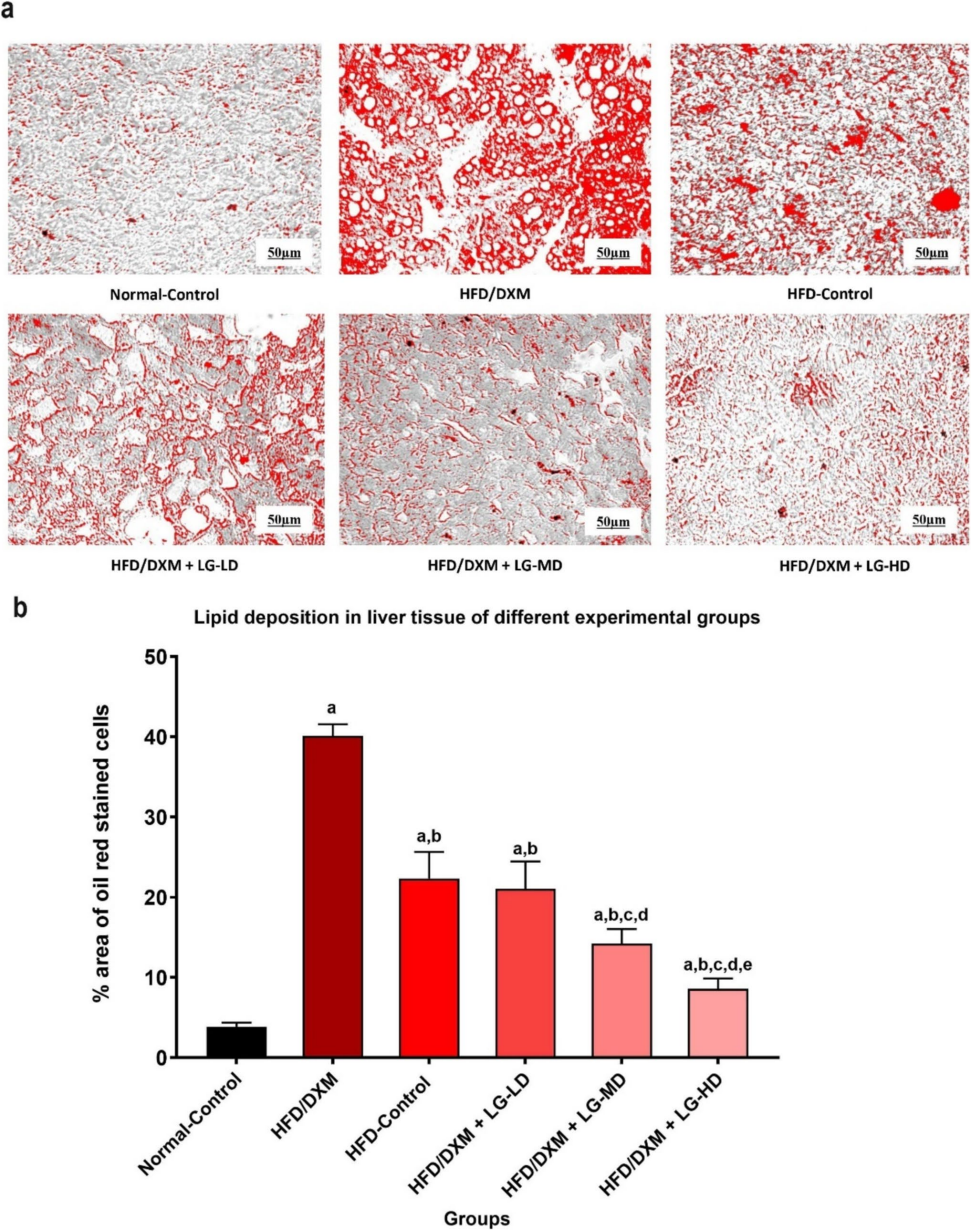
**a** Photomicrograph of liver tissue section stained with oil red in the different groups at the end of the experimental study. Images are captured with 20X magnification, and the scale bar is 50µm. The control group showed mild deposition of fat droplets. Withdrawal of DXM treatment after induction of steatosis in HFD-control group showed a moderate degree of fat droplet deposition. HFD/DXM-treated groups plus LG at different doses showed a lower degree of fat droplet deposition. **b** Statistically significant increase in lipid accumulation in DXM-treated group after 6 weeks compared to the control group, and groups treated with DXM plus LG at different doses (*P* < 0.05). ^“a,b,c,d,e”^ represents a significant difference (*P* < 0.05) from control, HFD/DXM, HFD-Control, HFD/DXM + LG-LD, and HFD/DXM + LG-MD, respectively. DXM, dexamethasone; HFD, high-fat diet; LG, liraglutide; LD, low-dose 0.2 mg/kg; MD, moderate-dose 0.4 mg/kg; HD, high-dose 0.8 mg/kg

A cardinal sign of fatty liver is the accumulation of lipid droplets in liver tissue, and therefore we assessed the accumulation of lipid droplets in liver tissues among different treatment groups using oil red O staining. We detected basal levels of oil red staining in the liver tissues of normal-control rats. On the contrary, DXM treatment resulted in a significantly increased in the % area of oil red stained cells in liver tissues compared to the control group (40.09±1.47 *vs* 3.83±0.53, *P* < 0.05). Although discontinuation of DXM treatment was associated with a decrease in lipid accumulation, this reduction did not reach to the normal level (40.09±1.47 *vs* 22.35±3.31). In contrast, coadministration of LG (0.2, 0.4, and 0.8 mg/kg) in DXM-treated rats was associated with dose dependent decrease in accumulation of lipid droplets compared to DXM-treated rats (21.05±3.4, 14.25±1.79, 8.59±1.3, respectively, vs 40.09±1.47, *P* < 0.05) (Fig. 4a, b). Collectively, these data further validate the establishment of our fatty liver model and add more credence regarding the beneficial role of LG on DXM-induced fatty liver.

### Liraglutide reduces liver inflammation-induced by hepatic lipid accumulation

**Fig. 5:**
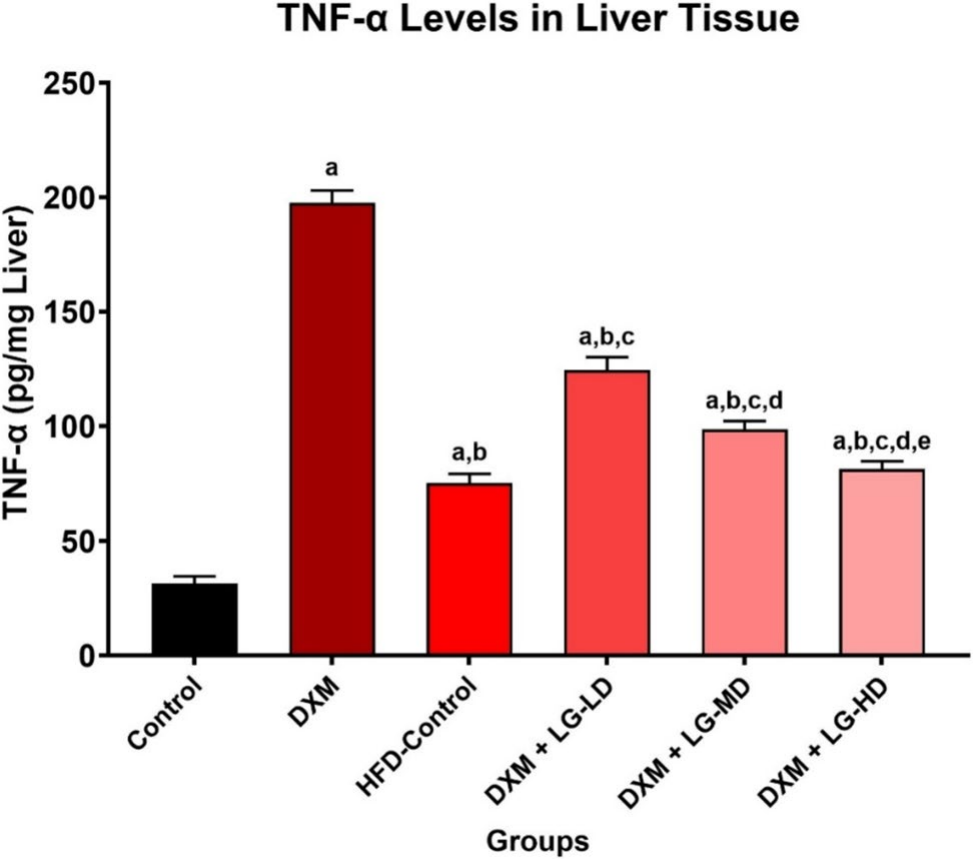
Statistically significant increase in tumor necrosis factor-α (TNF-α) levels in liver tissue in HFD/DXM-treated rats after 6 weeks compared with the normal-control group, HFD-control group and groups treated with HFD/DXM plus LG at different doses (*P* < 0.05). ^“a,b,c,d,e”^ represents a significant difference (*P* < 0.05) from control, HFD/DXM, HFD-Control, HFD/DXM + LG-LD, and HFD/DXM + LG-MD, respectively. Notably, TNF-α levels in the HFD/DXM + LG-HD group were not significantly lower than those in the HFD-Control group, indicating that high-dose liraglutide (0.8 mg/kg) protects against DXM-induced but not HFD-induced liver inflammation. DXM, dexamethasone; HFD, high-fat diet; LG, liraglutide; LD, low-dose 0.2 mg/kg; MD, moderate-dose 0.4 mg/kg; HD, high-dose 0.8 mg/kg

Inflammation is one of the most important factors in the pathogenesis of fatty liver disease. The contents of pro-inflammatory cytokine TNF-α in hepatic tissues were increased in DXM-treated group compared to the control group (197.58±5.32 *vs* 31.48±3.05, *P* < 0.05). In contrast, coadministration of LG (0.2, 0.4, and 0.8 mg/kg) in DXM-treated rats resulted in a dose-dependent decrease in TNF-α levels in hepatic tissues compared with rats treated with DXM alone (124.49±5.66, 98.61±3.57, 81.39±3.42, respectively, vs 197.58±5.32, *P* < 0.05). Interestingly, liver TNF-α levels in the HFD/DXM + LG-HD group were not significantly lower than those in the HFD-Control group (81.39±3.42 *vs* 75.28±4.03). This suggests that while high-dose liraglutide (0.8 mg/kg) protects against DXM-induced liver inflammation, it does not appear to counteract HFD-induced liver inflammation (Fig. 5).

### Glucocorticoids regulate FANS and CD36 genes

**Fig. 6:**
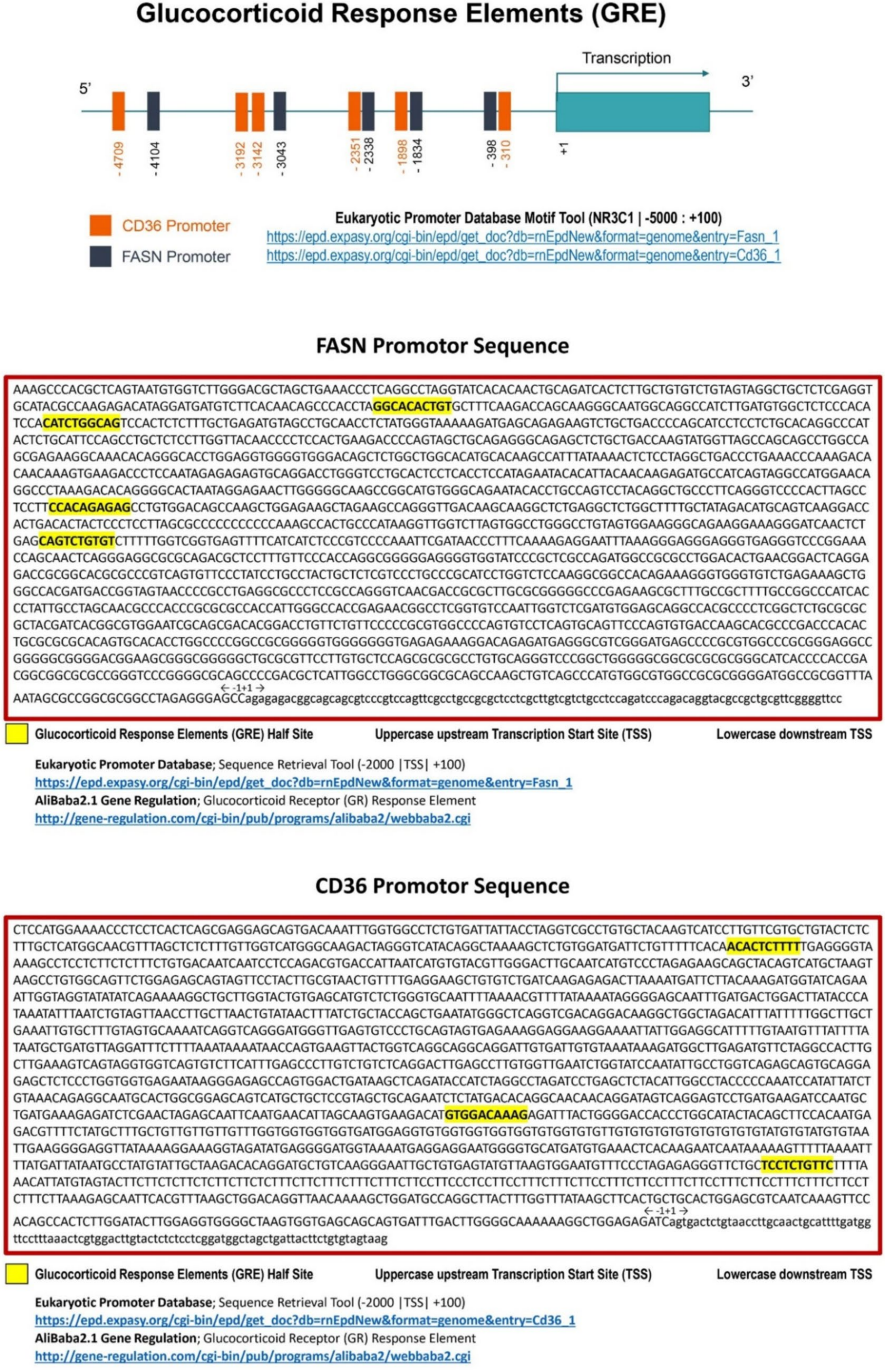
Locations of glucocorticoid response elements (GRE) identified in the promoter sequences of FASN and CD36 genes. The promoter regions analyzed span − 2000 to +100 relative to the transcription start site (TSS). GRE half-sites are highlighted in yellow, representing potential binding sites for glucocorticoid receptors. Promoter sequences were retrieved using the Eukaryotic Promoter Database (EPD), and GREs were predicted using the AliBaba2.1 tool. CD36, Cluster of differentiation 36; FASN, Fatty acid synthase

Given the fundamental role of glucocorticoid receptors in regulating the transcription of certain genes, we aimed to interrogate whether *FASN* and *CD36* genes are regulated by glucocorticoid receptors. Thus, we performed in silico analysis and revealed that the promotor regions of *FASN* and *CD36* genes, two genes important for lipid metabolism, contain several glucocorticoid response elements (Fig. 6). These data suggest that FASN and CD36 might be regulated by glucocorticoid receptors and such regulation may occur at transcriptional level.

The results revealed multiple GREs in the promoter regions of FASN and CD36, suggesting that these genes might be directly regulated by glucocorticoid receptors at the transcriptional level. These findings are summarized and visualized in Fig. 6, providing a detailed map of the GREs within the promoter sequences.

### Liraglutide diminishes the effects of DXM on the expression of FASN and CD36

**Fig. 7:**
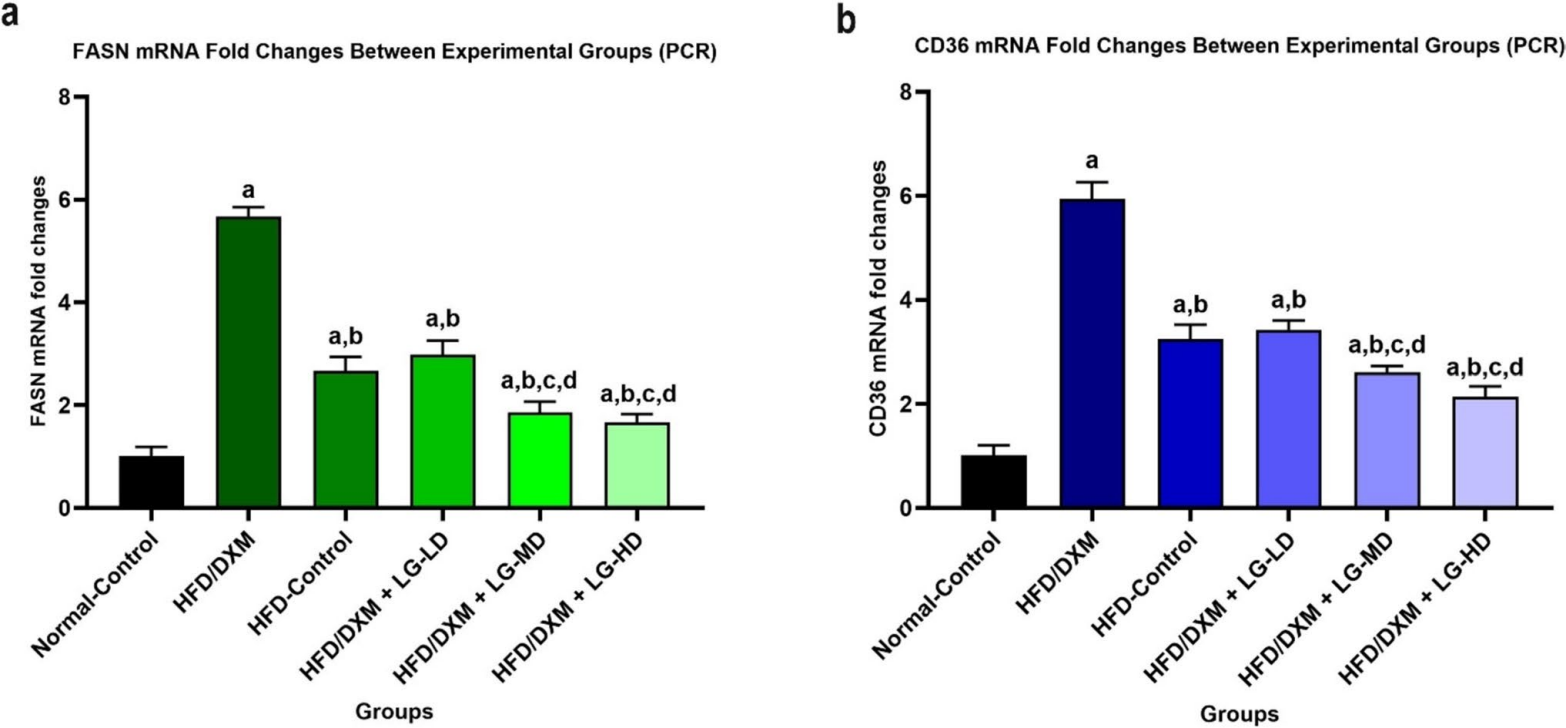
Statistically significant increase in FASN (**a**) and CD36 (**b**) mRNA in HFD/DXM-treated group compared with the normal-control group, and groups treated with HFD/DXM plus LG at different doses (*P* < 0.05). ^“a,b,c,d”^ represents a significant difference (*P* < 0.05) from Control, HFD/DXM, HFD-Control, and HFD/DXM + LG-LD, respectively. DXM, dexamethasone; HFD, high-fat diet; LG, liraglutide; LD, low-dose 0.2 mg/kg; MD, moderate-dose 0.4 mg/kg; HD, high-dose 0.8 mg/kg

To this end, our data demonstrated that DXM induces fatty liver and this effect is most likely associated with altered expression of *FASN* and *CD36*. To validate our in silico results, we investigated the effect of DXM and/or LG on FASN and CD36 at the DNA level using real-time quantitative reverse transcription PCR (RT-qPCR). PCR analysis revealed that the expression of *FASN* in the DXM-treated group was significantly higher compared to its level in the control group (5.67± 0.18 *vs* 1.0± 0.18, *P* < 0.05). Similarly, the expression of *CD36* in the DXM-treated group was markedly higher than in the control group (5.93± 0.32 *vs* 1.0±0.20, *P* < 0.05). Notably, LG treatment at three different doses (0.2, 0.4, 0.8 mg/kg/day) resulted in a remarkable reduction in *FASN* expression (2.97±0.27, 1.86±0.21, 1.66±0.16, *respectively*) relative to DXM-treated rats (5.67± 0.18, *P* < 0.05). Similarly, *CD36* expression in the DXM and LG co-treated rats (3.42±0.18, 2.60±0.12, 2.14±0.20, *respectively*) was significantly reduced compared with the rats treated with DXM alone (5.93± 0.32, *p* < 0.05) (Fig. 7a and b).

**Fig. 8:**
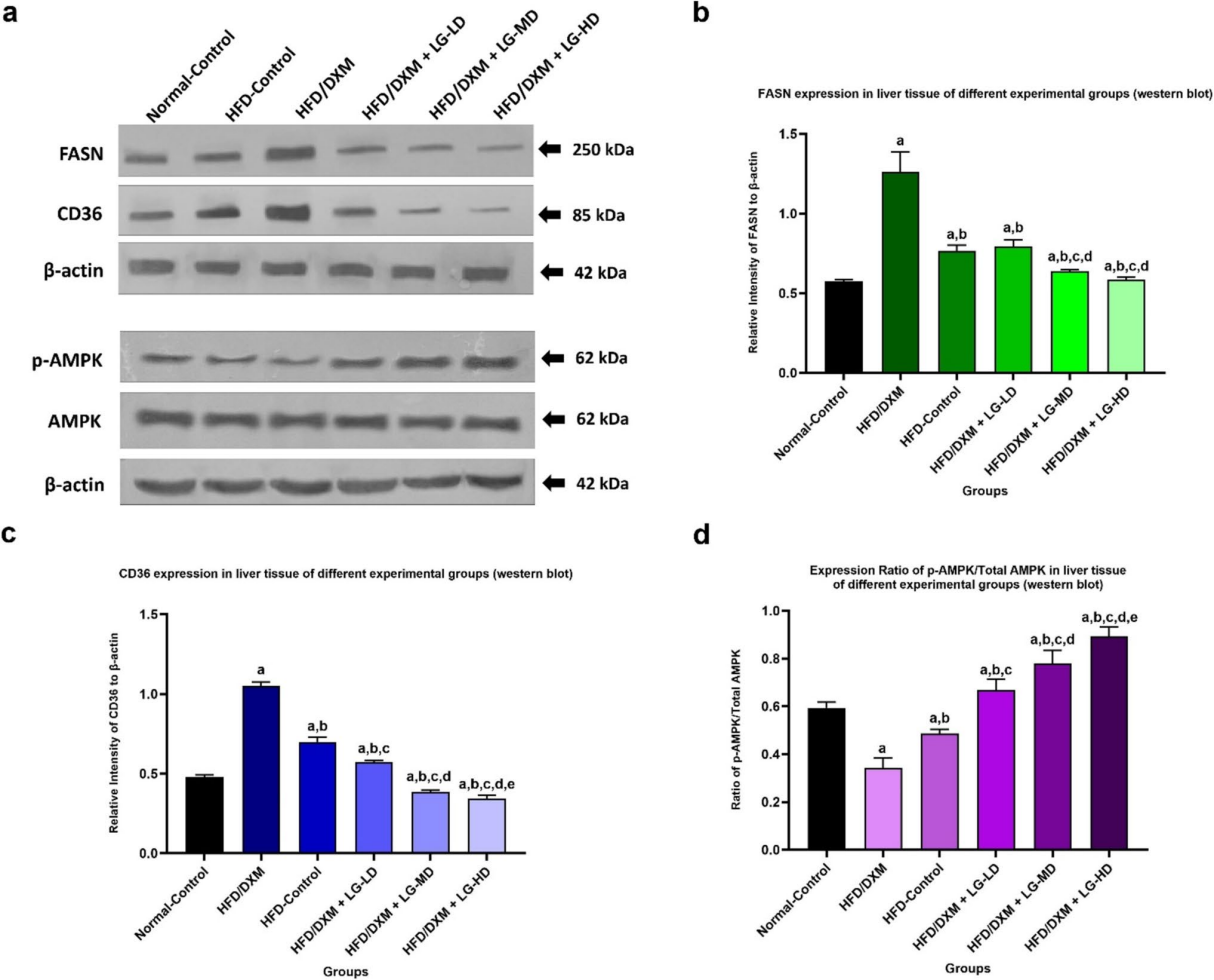
**a** Western blot of FASN, CD36, and p-AMPK expression in liver tissue in the different experimental groups at the end of the DXM and LG treatment period. The protein zone of FASN and CD36 is high in the HFD/DXM-treated group compared with the normal-control group, HFD-control group, and LG combined with DXM-treated groups. Reductions of FASN and CD36 protein expression in the groups treated with HFD/DXM coupled with LG at a high dose compared to mid-dose and low-dose LG. Notably, the levels of phosphorylated AMPK (p-AMPK) were reduced in the HFD/DXM-treated group compared with the normal control group, indicating that DXM suppresses the phosphorylation of AMPK. In contrast, LG-treated groups exhibited a dose-dependent increase in p-AMPK levels, suggesting that LG actively enhances AMPK phosphorylation, which may represent a mechanism by which LG suppresses FASN and CD36 expression. **b**,** c**,** d** Statistically significant increase in FASN (**b**) and CD36 (**c**) expression in the HFD/DXM-treated group compared to the normal-control group, HFD-control group, and HFD/DXM-treated groups plus LG at different doses (*P* < 0.05). A significant decrease in FASN (**b**) and CD36 (**c**) expression was observed in the group treated with HFD/DXM and high-dose LG compared with groups treated with HFD/DXM coupled with mid-dose and low-dose LG (*P* < 0.05). Additionally, p-AMPK levels (Fig. 8d) were significantly reduced in the HFD/DXM-treated group compared with the normal-control group. p-AMPK levels are significantly increased in groups treated with HFD/DXM coupled with LG in a dose-dependent manner compared to HFD/DXM-treated group (*P* < 0.05) ^“a,b,c,d,e”^ represents a significant difference (*P* < 0.05) from Control, HFD/DXM, HFD-Control, HFD/DXM + LG-LD, and HFD/DXM + LG-MD, respectively. AMPK, adenosine monophosphate-activated protein kinase; CD36, cluster of differentiation 36; DXM, dexamethasone; FASN, fatty acid synthase; HFD, high-fat diet; LG, liraglutide; LD, low-dose 0.2 mg/kg; MD, moderate-dose 0.4 mg/kg; HD, high-dose 0.8 mg/kg; p-AMPK, phosphorylated Adenosine Monophosphate-Activated Protein Kinase

Subsequently, to assess *FASN* and *CD36* expression at the protein level, we performed Western blot (WB) and immunohistochemical (IHC) analyses. Immunoblot data demonstrated that DXM treatment upregulated the protein expression levels of FASN and CD36 in liver tissue, an effect which was significantly reduced by cotreatment with LG in a dose-dependent manner. This reduction was further associated with changes in AMPK activation, where DXM was shown to suppress AMPK phosphorylation, while LG enhanced AMPK phosphorylation. These findings suggest that LG’s ability to activate AMPK phosphorylation could serve as a potential mechanism for suppressing the expression of *FASN* and *CD36* (Fig. 8a).

The WB analysis revealed that the expression of *FASN* in the DXM-treated group was significantly higher compared with its level in the control group (1.26±0.12 *vs* 0.58±0.01, *P* < 0.05). Similarly, the expression of *CD36* in the DXM-treated group was significantly greater than that detected in the control group (1.05±0.02 *vs* 0.48±0.01, *P* < 0.05). Also, the expression of *FASN* and *CD36* in the DXM-treated group was significantly higher than those detected in the DXM-withdrawal group (1.26±0.12, 1.05±0.02 *vs* 0.77±0.04, 0.70±0.03, *respectively*). Interestingly, LG treatment at three different doses (0.2, 0.4, 0.8 mg/kg/day) resulted in a remarkable reduction in FASN protein levels (0.79±0.04, 0.64±0.01, 0.59±0.01, respectively) compared with that of DXM-treated rats (1.26±0.12, *P* < 0.05). Similarly, the expression of *CD36* in the LG coadministered with DXM-treated rats (0.57±0.01, 0.39±0.01, 0.35±0.02, respectively) were significantly lower than the expression in rats received DXM alone (1.26±0.12, *p* < 0.05) (Fig. 8b, c).

To further elucidate the mechanism underlying this reduction, we investigated the activation of AMPK by assessing both total AMPK and its phosphorylated form (p-AMPK, Thr172). Our data demonstrated that DXM-treated group was significantly suppressed the phosphorylation of AMPK compared with the control group (0.34±0.04 *vs* 0.59±0.02, *P* < 0.05). Notably, LG treatment increase phosphorylation of AMPK compared with the DXM-treated group in a dose-dependent manner (0.67±0.05, 0.78±0.05, 0.89±0.04 *vs* 0.34±0.04, respectively) (Fig. 8d). This finding suggests that AMPK activation might play a key role in mediating the inhibitory effects of LG on FASN and CD36 expression.

Next, we performed Pearson’s correlation analysis and observed that there is a positive correlation between FASN or CD36 protein expression and the extent of lipid accumulation in liver tissues in groups treated with DXM + LG doses (Pearson’s Correlation Coefficient (r) between 0 and 1) (Supplementary fig. 1).

**Fig. 9:**
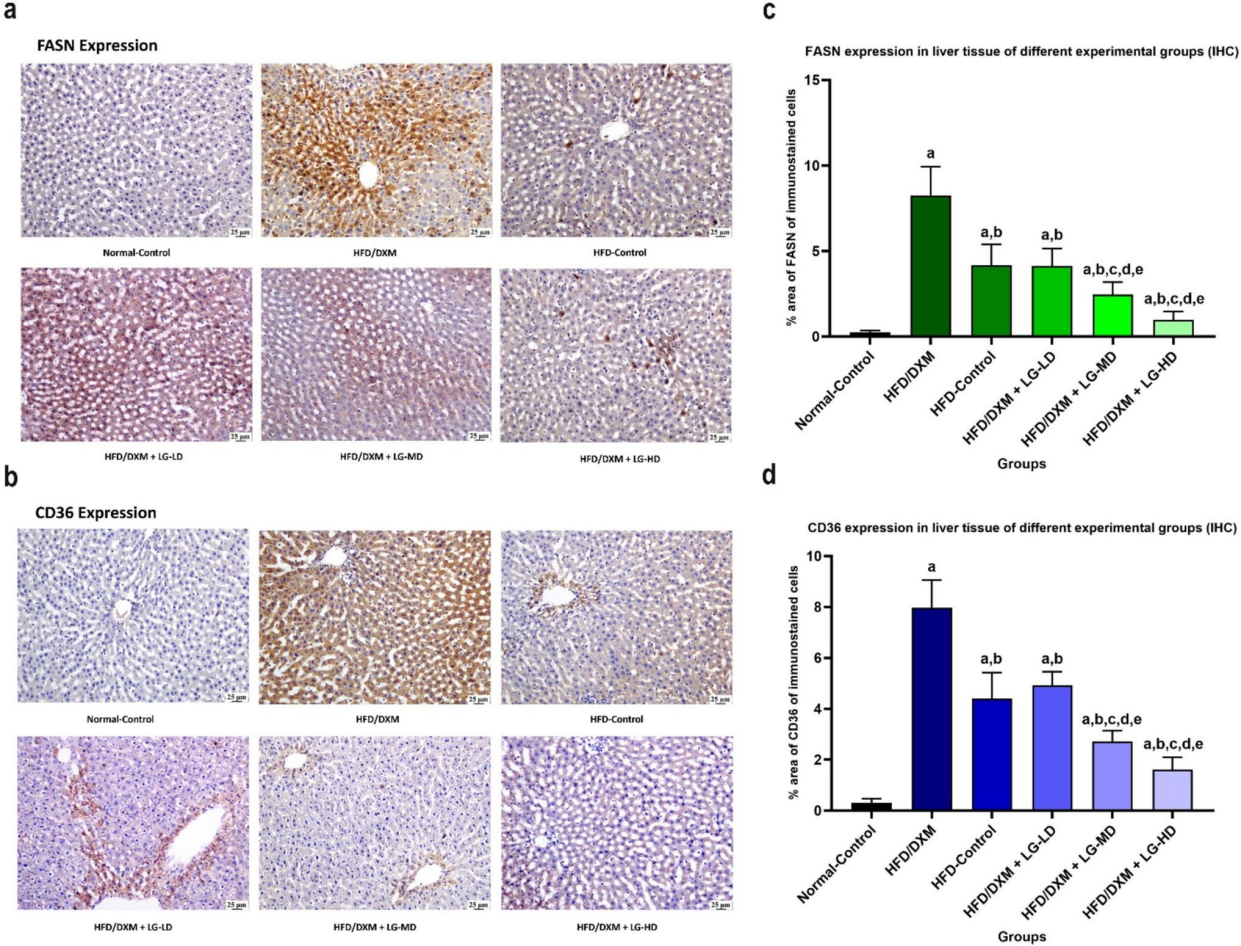
**a**, **b** Photomicrograph of liver tissue section stained with immunostaining of FASN (Fig. 8a) and CD36 (Fig. 8b) in the different groups at the end of the experimental study. Images are captured with high magnification, and the scale bar is 25µm. The normal-control group showed weak expression of FASN and CD36. Withdrawal of DXM treatment after induction of steatosis in HFD-control group showed a moderate degree of FASN and CD36 expression. HFD/DXM coupled with LG at different doses showed less FASN and CD36 expression compared to the HFD/DXM-treated group. **c**, **d** Statistically significant increase in FASN expression (Fig. 8c) and CD36 expression (Fig. 8d) in HFD/DXM-treated group compared to the normal-control group, HFD-control group, and groups treated with HFD/DXM plus LG at three different doses (*P* < 0.05). ^“a,b,c,d,e”^ represents a significant difference (*P* < 0.05) from Control, HFD/DXM, HFD-Control, HFD/DXM + LG-LD, and HFD/DXM + LG-MD, respectively. DXM, dexamethasone; HFD, high-fat diet; LG, liraglutide; LD, low-dose 0.2 mg/kg; MD, moderate-dose 0.4 mg/kg; HD, high-dose 0.8 mg/kg

This finding is further confirmed at the histological level. As indicated in Fig. 9a and b, treatment with DXM upregulates *FASN* and *CD36* in liver tissue, an effect significantly reduced by cotreatment with LG in a dose-dependent manner. Our data showed that the immunoreactivity of FASN in the rats treated with DXM was significantly higher than that in the control group (8.27±1.66 *vs* 0.25± 0.11, *P* < 0.05). Parallelly, the intensity of CD36 in rats treated with DXM showed significantly higher expression of this protein compared with its level in the control group (7.98±1.09 *vs* 0.31±0.16, *P* < 0.05). Also, the expression of *FASN* and *CD36* in the DXM-treated group was significantly higher than those detected in the DXM-withdrawal group (8.27±1.66 and 7.98±1.09, *vs* 4.17±1.23 and 4.40±1.03, respectively, *P* < 0.05). On the other hand, the amount of *FASN* expression in the groups given DXM and LG was notably reduced compared with the DXM-treated group, with the reduction correlating to the dosage of LG (4.14±1.02, 2.46±0.73 and 0.98±0.48, respectively, vs 8.27±1.66, *P* < 0.05). Additionally, levels of CD36 were notably decreased in groups treated with different doses of DXM and LG, in a manner dependent on the dose of LG, when compared with animals treated DXM (4.93±0.53, 2.72±0.43 and 1.62±0.48, respectively, vs 7.98±1.09, *P* < 0.05) (Fig. 9c, d).

Importantly, statistical analysis confirmed a positive correlation between liver tissue FASN, CD36 immunoreactivity expression, and lipid accumulation in groups treated with DXM and LG at different doses (Pearson’s correlation coefficient (*r*) between 0 and 1) (Supplementary fig. 2).

## Discussion

Despite the pleiotropic clinical benefits of glucocorticoids, chronic use of glucocorticoids is associated with metabolic side effects including insulin resistance, central obesity, dyslipidemia, and fatty liver (Rahimi et al. 2020). These undesirable effects may reduce glucocorticoids tolerability and limit their use. Thus, identifying a potential therapeutic strategy to hamper the development of glucocorticoids-induced fatty liver is an unmet clinical need. The current study was designed to explore a clinically relevant therapy that ameliorates DXM-induced fatty liver and other metabolic disturbance. Our findings revealed that DXM treatment induces fatty liver as evidenced by increased total body weight, alterations in macroscopic and histological features of livers, and disturbance in biochemical parameters. Interestingly, liraglutide (LG) treatment reversed DXM-induced fatty liver and this effect seems to be dose-dependent. Mechanistically, DXM triggers the protein expression of FASN and CD36, two essential proteins for fatty acids synthesis and uptake, and LG treatment mitigates DXM-induced upregulation of FASN and CD36 in a dose-dependent manner.

Chronic use of glucocorticoids is associated with the derangement of lipid metabolism and the development of NAFLD. Nevertheless, the molecular mechanisms underlying glucocorticoid-induced NAFLD is not completely understood (Jarmakiewicz-Czaja et al. 2022). Thus, in the current study, we attempted to explore how glucocorticoids alter lipid metabolism at the molecular level and examine the potential impact of GLP-1 agonist, LG, in the treatment of DXM-induced NAFLD. Interestingly, our computational analysis revealed that the promotor regions of the two genes, *FASN* and *CD36*, harbor several glucocorticoid response elements, suggesting that these genes might be regulated by DXM at the transcriptional levels (Fig. 6). This notion is substantiated by a previous finding that the glucocorticoid receptor (GR) binds to specific nucleotide invariant sequences in the DNA promoter/enhancer called glucocorticoid response element, which is comprised of two half-sites (AGAACA) separated by a three-base-pair spacer (Weikum et al. 2017). This binding either activates or represses transcription according to the binding to the general transcription factors, which is partly mediated by co-activators and/or co-repressors, respectively (Parsonnet et al. 2019). Furthermore, it has been reported that DXM induces NAFLD through promoting FASN and CD36 expression, hepatic DNL pathway, FAs transport, and lipid accumulation (Woods et al. 2015). These reports add credence to and support our findings. Our data revealed that DXM treatment increases the total body weight of rats and this effect could be explained by exacerbated accumulation of lipids. This finding is in line with previously published data that glucocorticoids increase body weight and lipid accumulation in animals. Increased body weight is sometimes associated with the development of fatty liver, which is a hallmark of NAFLD (Akalestou et al. 2020).

Our results showed that DXM treatment increases ALT, AST, FBS, TC, LDL-C, VLDL-C, and TG, while the level of HDL decreased. This finding is substantiated by a previous study showing that the typical findings in NAFLD are the elevation in e ALT and AST (Iser and Ryan 2013). Additionally, other studies indicated a significant relationship between the presence of steatohepatitis and increased levels of ALT and AST, and these biomarkers have been considered as a part of some steatohepatitis diagnostic panels (de Alwis and Day 2007; Neuschwander‐Tetri et al. 2010; Ratziu et al. 2000). Hasona and Morsi showed that the injection of DXM results in an increase of TC, LDL-C, and TG with a reduction of HDL-C in the blood serum of adult male rats (Hasona and Morsi 2019). In an independent study, Ensler et al. proved that DXM increases the level of VLDL-C through an indirect stimulatory effect mediated via GR on VLDL-C receptor gene transcription activation (Ensler et al. 2002). These data are in accordance with our data that DXM-induced change in body weight and fat accumulation in the liver is reversible, and the withdrawal of DXM after 3 weeks of treatment resulted in a return of the body weight, ALT, AST, FBS, TC, LDL-C, VLDL-C, TG, and HDL to a comparable level to the control group.

At the histological levels, DXM treatment resulted in diffuse hepatocellular vacuolation, marked mononuclear inflammatory cells infiltrating hepatic parenchyma with hepatocellular necrosis, and Kupffer cells hyperplasia. Interestingly, these histopathological changes were combined with the accumulation of fat droplets in liver tissue. This finding follows previously published reports that vacuolation and ballooning of hepatic cells were observed in the liver of the DXM-treated rabbits and were associated with dose- and duration-related degenerative changes in these cells. Dilatation and congestion of the central hepatic vein and sinusoidal capillaries were also observed in DXM-treated animals (Jasim et al. 2022).

The accumulation of fat droplets in glucocorticoid-treated animals can also be explained by glucocorticoid-induced upregulation of FASN and CD36. It is well known that the effect of glucocorticoid is mediated through the binding of glucocorticoid with its cognate receptor with subsequent translocation into the nucleus, whereby it regulates gene expression (Woods et al. 2015). Intuitively, it is anticipated that blocking glucocorticoid receptor could be a beneficial treatment of glucocorticoid-induced NAFLD. Unfortunately, there is no selective glucocorticoid receptor antagonist available for clinical use. In addition, even the clinical use of an available non-selective glucocorticoid receptor antagonist (Mifepristone) is associated with serious side effects. Furthermore, the clinical benefit of glucocorticoid receptor antagonist will interfere with the clinically desirable action of glucocorticoids (Kroon et al. 2018). Therefore, we aimed to test an alternative approach that blocks glucocorticoid-induced fatty liver without compromising its beneficial action, given the fact that upregulation of FASN and CD36 is crucial in mediating glucocorticoid-induced fatty liver.

Notably, liraglutide (LG), a glucagon-like peptide 1 (GLP-1) analog, is one of a newly emerged class of anti-obesity drugs that exerts a beneficial effect on obesity and NAFLD (Fang et al. 2020). In this study, we investigated whether LG could ameliorate glucocorticoid-induced NAFLD. As expected, LG treatment significantly and dose-dependently ameliorated DXM-induced fatty liver and abrogated DXM-induced upregulation of FASN and CD36 protein expression. Chen et al. observed that FASN expression was downregulated in LG-treated mice in perirenal, epididymal, and visceral adipose tissues in a dose-dependent and diet-independent manner compared to control mice. The authors concluded that LG could reduce FASN expression in adipocytes by activating many proteins implicated in adipocytic differentiation and lipid metabolism including protein kinase A (PKA), mitogen-activated protein kinase (MAPKs), phosphorylation of cAMP response element binding protein (pCREB), and extracellular signal-regulated kinase (ERKs) in a dose- and time-course-dependent manner (Chen et al. 2017). In an independent study, Nagashima et al. demonstrated that administration of GLP-1 analogue significantly suppressed atherosclerotic lesions and macrophage infiltration in the aortic wall, associated with significant decreases in foam cell formation and downregulation of CD36 in macrophages, and this effect was abolished by pretreatment with exendin, a specific GLP-1 antagonist (Nagashima et al. 2011). Kong et al. showed that intracellular TG content was positively correlated with the expression of CD36. Furthermore, LG treatment reduced intracellular lipid deposition, improved lipid metabolism, and downregulated the expression of CD36 in myoblasts (Kong et al. 2022).

**Fig. 10:**
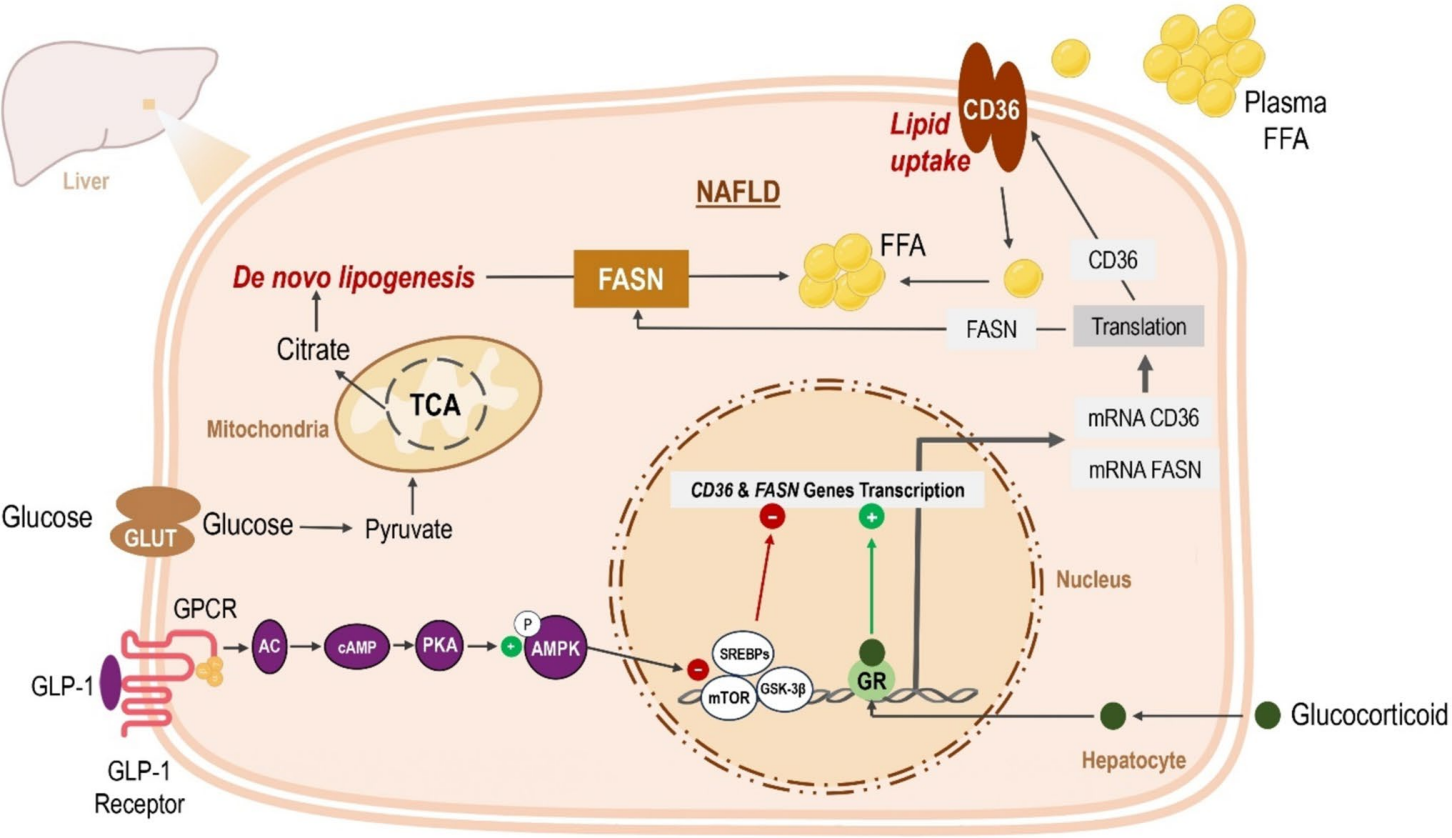
Effect of glucocorticoids and/or GLP-1 agonist on lipid metabolism. Glucocorticoids increase gene expression of FASN, which plays a key role in de novo fatty acid synthesis, and CD36, which is responsible for lipid uptake. Stimulation of the GLP-1 receptor (GLP-1R) leads to an increase in intracellular cAMP, which activates protein kinase A (PKA). PKA, in turn, phosphorylates and activates AMP-activated protein kinase (AMPK), a central metabolic regulator. AMPK activation suppresses lipid synthesis and modulates gene expression, potentially contributing to the observed downregulation of FASN and CD36 in response to GLP-1 receptor stimulation. AC, adenylate cyclase; cAMP, cyclic AMP; AMPK, adenosine monophosphate-activated protein kinase; CD36, glycoprotein cluster of differentiation36; FASN, fatty acid synthase enzyme; FFA, free fatty acids; GPCR, G protein-coupled receptor; GR, glucocorticoid receptor; GSK-3B, glycogen synthase 3 beta; mTOR, mammalian target of rapamycin; NAFLD, non-alcoholic fatty liver disease; PKA, protein kinase A; SREBPs, sterol regulatory element-binding proteins; TCA, tricarboxylic acid cycle

To further elucidate the mechanism underlying the effects of LG on DXM-induced fatty liver, we explored the role of AMP-activated protein kinase (AMPK) as a potential regulator of FASN and CD36 expression. Western blot analysis demonstrated that DXM treatment significantly reduced AMPK phosphorylation (p-AMPK), indicating suppressed p-AMPK levels in the liver. These findings align with previous studies showing that AMPK activation is a key downstream pathway of GLP-1 receptor signaling that suppresses lipid synthesis (Li et al. 2016). Furthermore, AMPK phosphorylation has been shown to modulate downstream signaling pathways, including p38-MAPK, which contributes to the regulation of lipid metabolism and inflammation (Yang et al. 2020). Our results suggest that the beneficial effects of LG on DXM-induced fatty liver are mediated, at least in part, by AMPK activation, which in turn downregulates FASN and CD36 expression. Taken together, we speculate that LG attenuates DXM-induced fatty liver, at least in part, by downregulating the expression of CD36 and FASN. GLP-1 analogs have been shown to regulate multiple upstream signaling pathways that orchestrate FASN and CD36 expression independent on glucocorticoid receptors. In this context, activation of GLP-1 receptors by liraglutide may lead to activation of pleiotropic signaling cascades including, but not limited to, mammalian target of rapamycin (mTOR), glycogen synthase kinase 3 (GSK-3), and sterol regulatory element-binding proteins (SREBPs) which are known to be downstream signaling molecules to AMPK (Athauda and Foltynie 2016) (Fig. 10). Based on these data, we cannot rule out the possible modulatory effect of LG on any of these upstream molecular pathways that regulate FASN and CD36 expression; LG could repress FASN and CD36 expression either directly or indirectly via tuning any of those upstream mediators.

To this end, the current study revealed that FASN and CD36 genes harbor several glucocorticoid response elements in their promotor regions. Furthermore, the effect of glucocorticoids-induced FANS and CD36 upregulation and lipid accumulation in the liver can be ameliorated by the liraglutide treatment through pleiotropic signaling pathways. Since liraglutide is clinically used and has tolerable side effects profile, the use of LG to prevent glucocorticoid-induced fatty liver is a clinically feasible strategy. Future clinical studies are warranted to investigate the clinical efficacy of LG in the prevention and/or treatment of fatty liver disease.

## Summary and conclusion

Long-term therapy with DXM is implicated in NAFLD through upregulation of FASN and CD36 expression. The upregulation of FASN and CD36 is attributed to the presence of glucocorticoid response elements in their promotors. Increased FASN and CD36 expression in liver tissue upon DXM treatment resulted in altered serum liver enzymes and lipid profile, hepatic tissue architecture, and increased lipid accumulation. Most importantly, treatment with LG ameliorated DXM-induced NAFLD, and abrogated DXM-induced dysregulation of FASN, CD36, liver enzymes, and lipid profile. Collectively, these data suggest that LG might represent a potential treatment option for glucocorticoid-induced NAFLD.

## Supplementary Information

Below is the link to the electronic supplementary material.

Supplementary file1 (JPG 649 KB)

Supplementary file2 (JPG 700 KB)

Supplementary file3 (PDF 697 KB)

## Data Availability

All source data for this work (or generated in this study) are available upon reasonable request.
