# Supplementary figures and images for: Liraglutide mitigates dexamethasone-induced fatty acid synthase (FASN) and the cluster of differentiation36 (CD36) expression: a potential treatment for glucocorticoid-induced non-alcoholic fatty liver disease (NAFLD)
Source: Naunyn Schmiedebergs Arch Pharmacol. 2025 Jan 17;398(7):8567–85. doi: 10.1007/s00210-025-03789-6 (PMC12263778; doi:10.1007/s00210-025-03789-6)

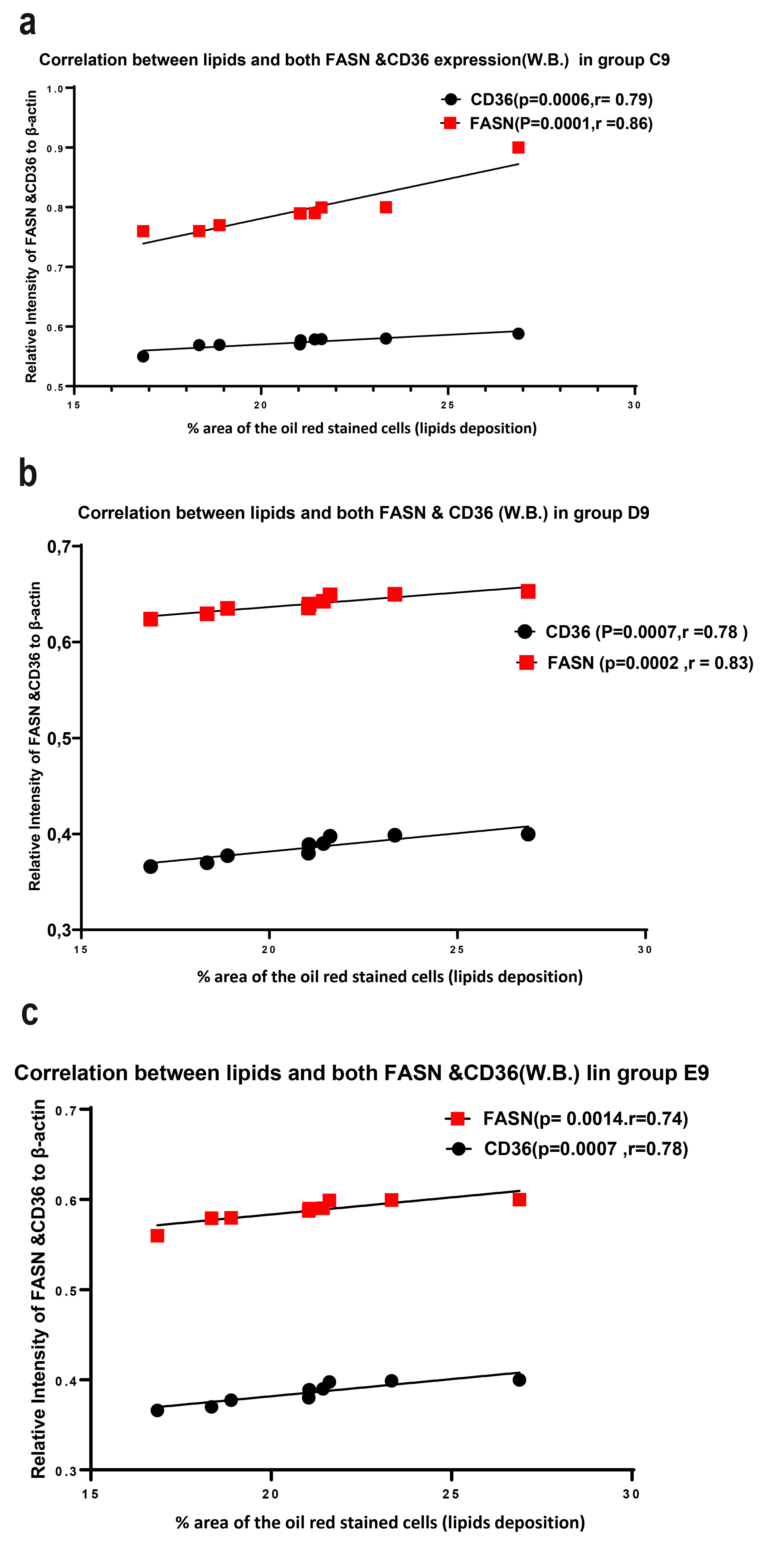

Supplement: Supplementary file 1 — Supplementary file1 (JPG 649 KB) [file 210_2025_3789_MOESM1_ESM.jpg]

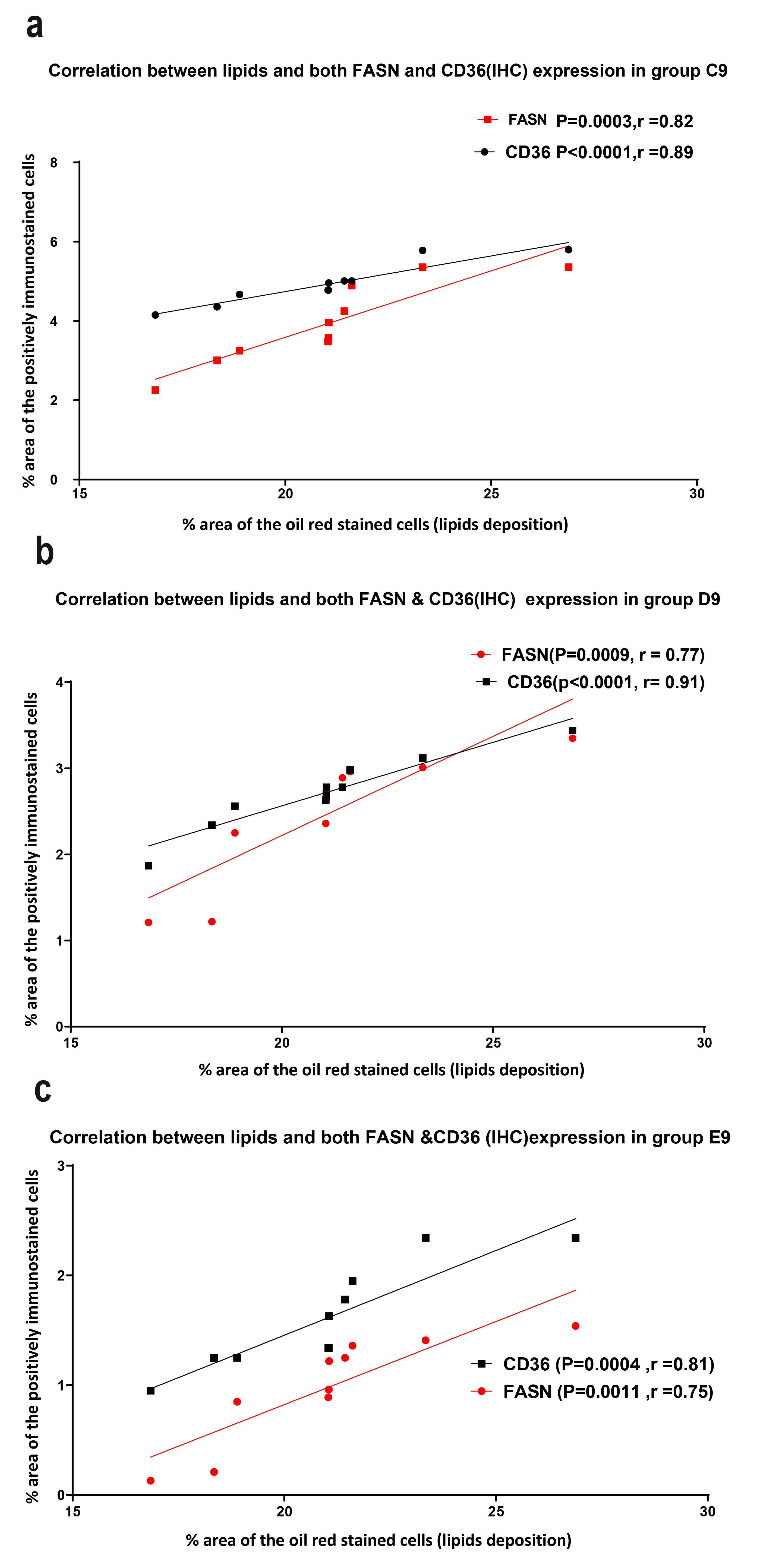

Supplement: Supplementary file 2 — Supplementary file2 (JPG 700 KB) [file 210_2025_3789_MOESM2_ESM.jpg]

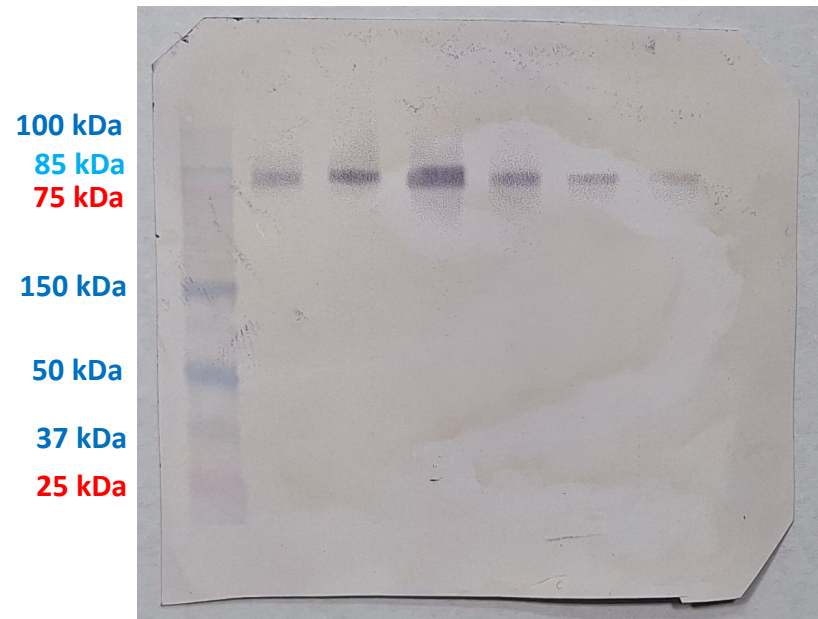

**CD36**

250 kDa  
150 kDa  
100 kDa  
75 kDa

50 kDa

42 kDa

37 kDa  
25 kDa

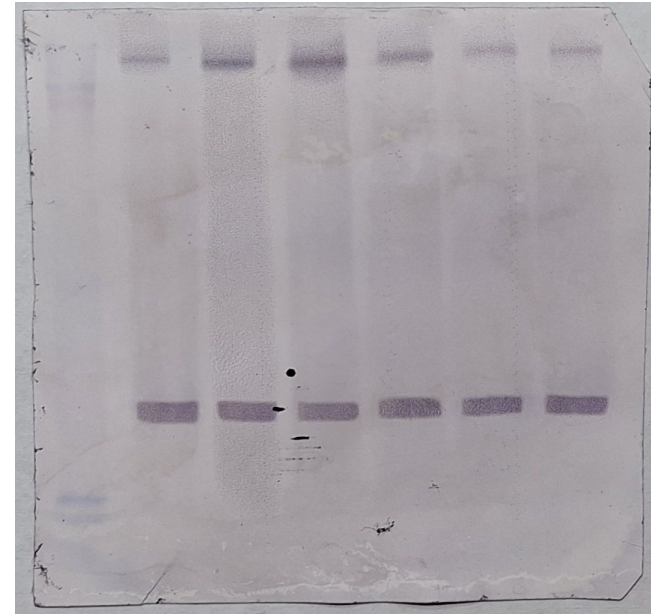

**FASN**

**$\beta$ -actin**

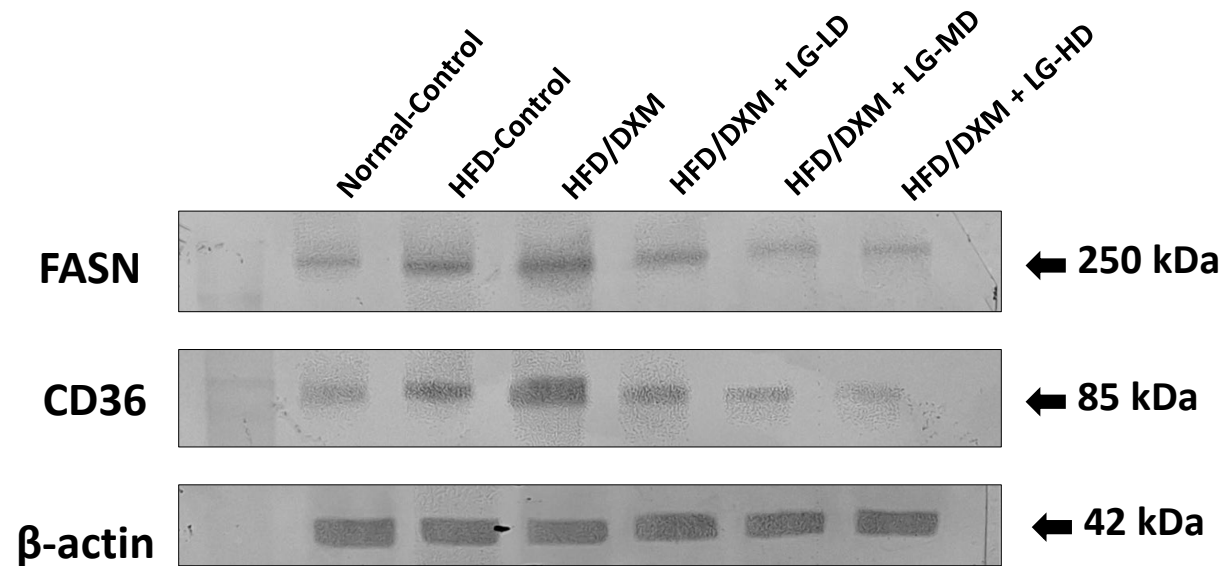

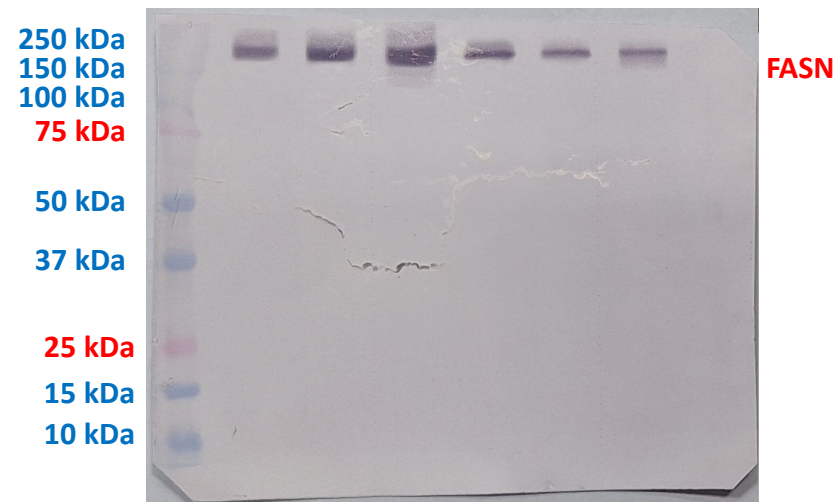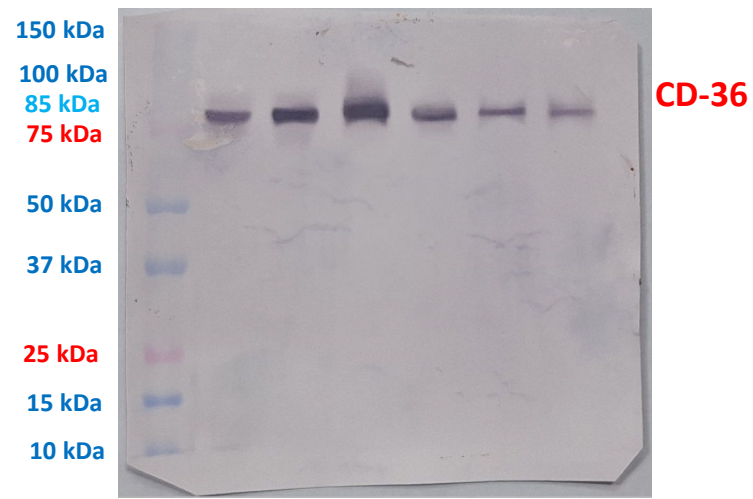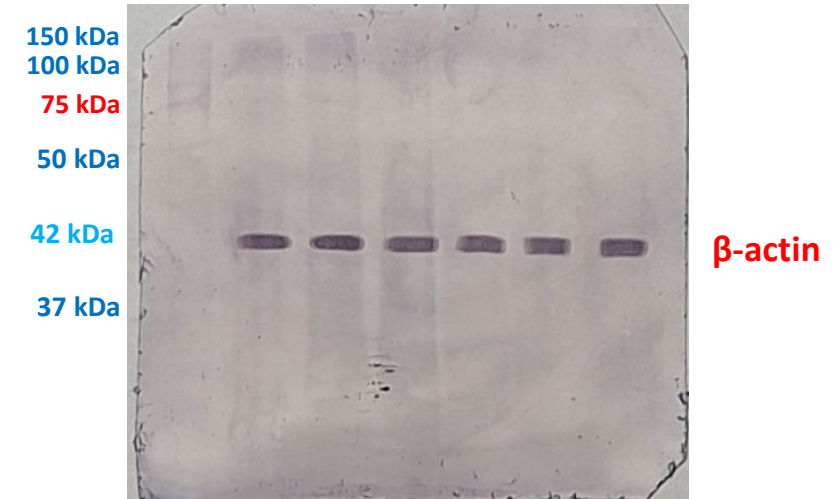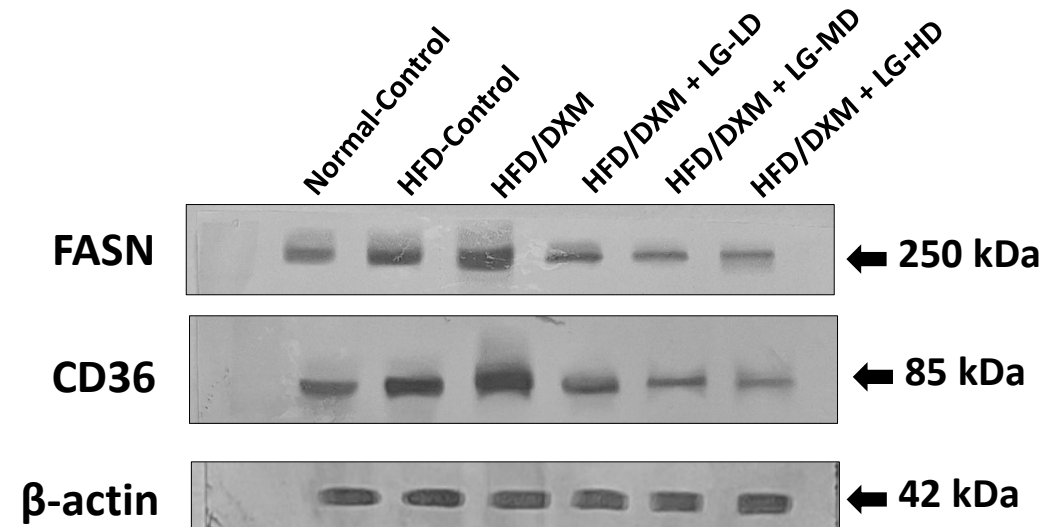

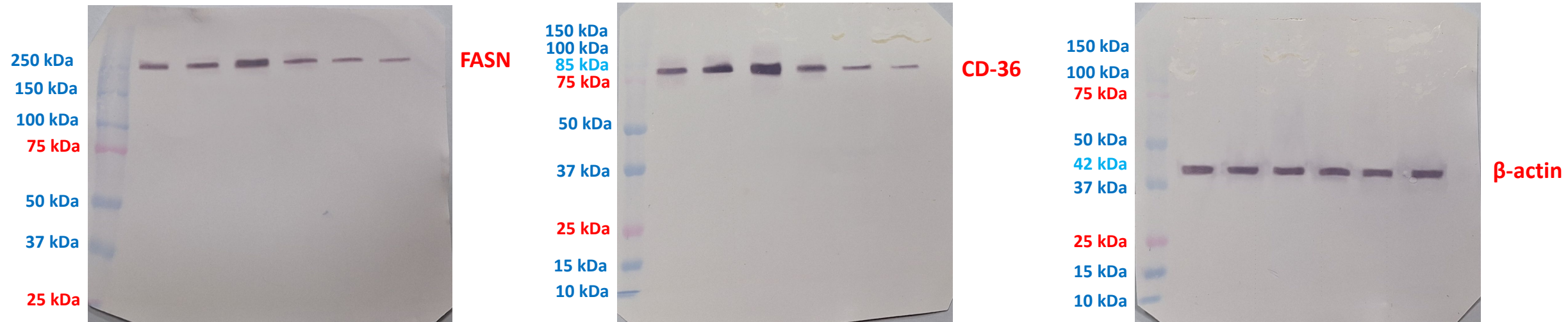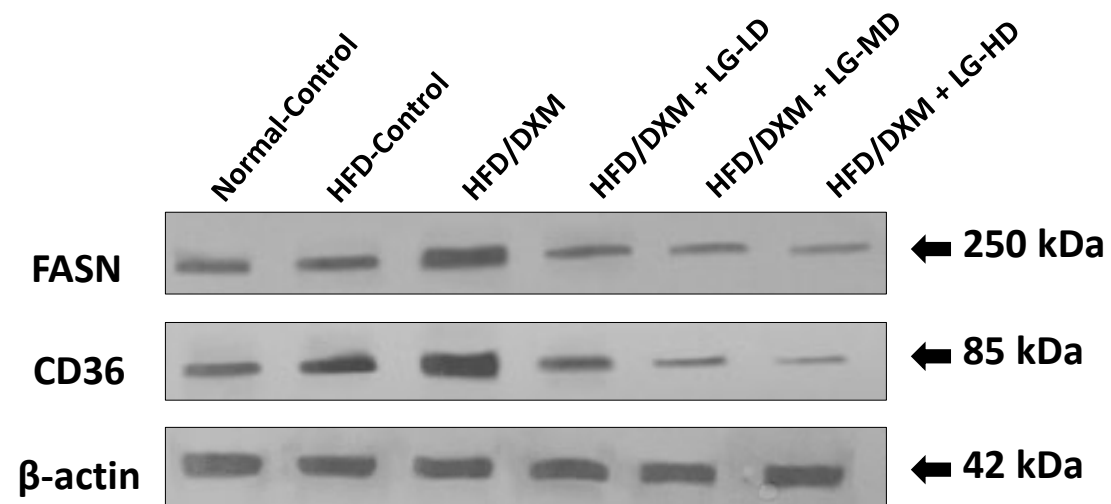

Supplement: Supplementary file 3 — Supplementary file3 (PDF 697 KB) [file 210_2025_3789_MOESM3_ESM.pdf]
